# Spatial data intelligence and city metaverse: A review

**DOI:** 10.1016/j.fmre.2023.10.014

**Published:** 2023-12-28

**Authors:** Xiaofeng Meng, Yong Li, Ke Liu, Yu Liu, Bin Yang, Xuan Song, Guoqiong Liao, Senzhang Wang, Ziqiang Yu, Longbiao Chen, Xiao Pan, Yuming Lin

**Affiliations:** a School of Information, Renmin University of China, Beijing 100872, China; b Department of Electronic Engineering, Tsinghua University, Beijing 100084, China; c Department of Information Sciences, National Natural Science Foundation of China, Beijing 100085, China; d Institute of Remote Sensing and Geographic Information Systems, School of Earth and Space Sciences, Peking University, Beijing 100871, China; e School of Data Science and Engineering, East China Normal University, Shanghai 200062, China; f Department of Computer Science and Engineering, Southern University of Science and Technology, Shenzhen 518055, China; g School of Information Management, Jiangxi University of Finance and Economics, Nanchang 330013, China; h School of Computer Science and Engineering, Central South University, Changsha 410017, China; i School of Computer and Control Engineering, Yantai University, Yantai 264005, China; j Department of Computer Science, Xiamen University, Xiamen 361000, China; k College of Information Science and Technology, Shijiazhuang Tiedao University, Shijiazhuang 050043, China

**Keywords:** Spatial data intelligence, City metaverse, Virtual-real interaction, Application prospect, Smart city

## Abstract

Spatial Data Intelligence (SDI) encompasses acquiring, storing, analyzing, mining, and visualizing spatial data to gain insights into the physical world and uncover valuable knowledge. These understandings and knowledge play a crucial role in connecting physical and virtual realms, such as in developing a City Metaverse (CM) aimed at enhancing and optimizing modern urban environments. The advancement of CM holds immense potential to benefit urban dwellers, making research on SDI an increasingly prominent area of focus. This paper contributes significantly by organizing the relevant research and technologies within a coherent framework. Firstly, we identify SDI technologies capable of collecting real-world information to construct a virtual CM. Subsequently, we delve into the technologies that can be compositely integrated with SDI to facilitate interaction with and management of actual cities from the virtual perspective. Additionally, we emphasize the effectiveness and potential of these methods in practical applications. Lastly, we conclude our survey by discussing emerging challenges associated with technological progress, the industrial chain, legal and regulatory aspects, and ethical and moral considerations.

## Abstract (author highlights)

• Developed a new framework clarifies virtual-real urban interactions, aiding understanding of urban space dynamics.
• Analyzes Spatial Data Intelligence and City Metaverse, offering insights into current status and future prospects.
• Shows mutual benefits: SDI aids CM growth and CM empowers SDI with virtual platforms for effective real-world interactions.

## Graphical abstract

**Figure:**
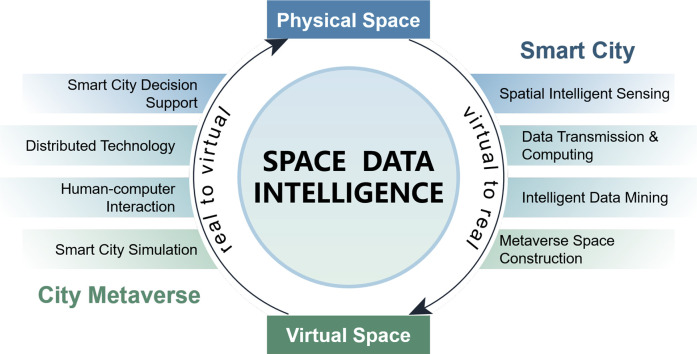

## 1 Introduction

Spatial data [1] refers to the information that characterizes individuals, objects, and events within natural geographical and human activity spaces. By primarily considering spatial data, the advancement of artificial intelligence (AI) technology across algorithms, data processing, and computing has contributed to spatial data collection and analysis, culminating in the emergence of Spatial Data Intelligence (SDI). SDI involves collecting, storing, analyzing, mining, and visualizing spatial data to grasp spatial information and reveal essential insights. Consequently, the fundamental technologies of SDI encompass spatial sensing, data storage, mining, and computing. The accumulation and digital storage of the spatial data, ranging from geo-location and terrain data to meteorological, population, and socioeconomic data, have amplified the significance of SDI in comprehending the world we inhabit.

On the other hand, the City Metaverse (CM) encompasses applying the Metaverse concept specifically within urban contexts [2]. The CM can be described as a virtual and digital urban space that incorporates urban data, models, and algorithms, enabling the simulation of diverse scenarios and synchronous interaction, also leverages various technologies including AI, cloud computing, big data, blockchain, and virtual reality (VR). CM exhibits distinctive characteristics such as being data-driven, facilitating authentic simulations, enabling intelligent decision-making, combining virtual and real elements, supporting human-computer interaction, spanning multiple domains, and prioritizing data security [3]. With such multidimensional capabilities, the CM demonstrates the vast potential in diverse applications such as urban planning, traffic management, cultural tourism, urban management, anticipating smart cities, optimizing urban resource utilization, and bolstering the sustainability of urban life.

The rapid advancement in AI has sparked a notable collaboration and interdependence between SDI and CM, resulting in a synergistic relationship between the virtual and real domains. On one hand, SDI catalyzes various technologies, establishing a pipeline to utilize spatio-temporal data through the “sensing-calculation-mining-construction”. In essence, this process can be perceived as a continuous abstraction from the “real” to the “virtual” domain. On the other hand, the CM originates from virtual city simulations and digital properties using technologies such as VR, augmented reality (AR), and other synchronous interactions. These technologies facilitate the connection of physical space components through distributed systems and ultimately influence the physical world through intelligent decision-making support. This represents a process that moves from the “virtual” to the “real” domain.

Despite the significant volume of research conducted in SDI, CM, and associated technologies, there remains a notable gap in comprehensive surveys that effectively bridge the “virtual-real” connection between these domains. Consequently, this review aims to fill this void by establishing a theoretical framework that elucidates the interaction between “virtual” and “real”. Additionally, this review seeks to summarize the current status of core technologies in both SDI and CM, providing a holistic understanding of recent advancements. Moreover, the practical application prospects and future challenges associated will be analyzed, offering insights into their potential implementation and identifying areas that require further investigation. This review will provide valuable guidance for researchers and practitioners to develop SDI and CM technologies in real-world scenarios.

This review will be organized as follows: Section 2 will introduce the framework and the method used in the survey. Section 3 would summarize key SDI technologies to fulfill the “real-to-virtual”, while Section 4 would review the key technologies to support CM for “virtual-to-real” invention. After reviewing the key technologies, Section 5 will illustrate the current and potential collaboration of SDI and CM, and Section 6 will look into the future direction and risks. Section 7 will be a brief conclusion.

## 2 Framework and method

### 2.1 Theoretical framework and content

**Fig. 1:**
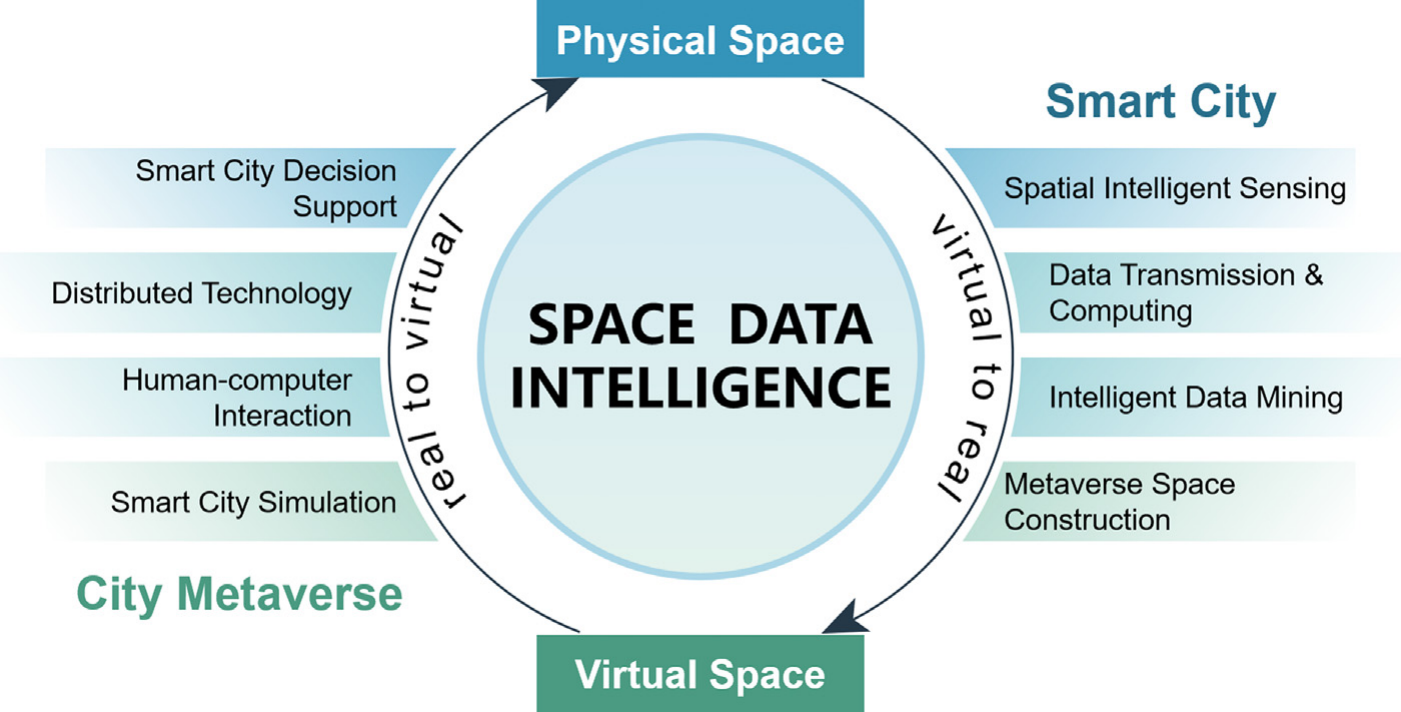
**The conceptual connection between SDI and SM**.

As previously discussed, SDI is rooted in the physical world, while CM is centered on the digital space. Together, they create a synergistic relationship that merges the virtual and real domains. This synergy has inspired the development of a theoretical framework, as depicted in Fig. 1. Within this framework, SDI plays a crucial role as a key technology and bridge, empowering the construction of the CM within the digital space.

In this review, our focus revolves around the fundamental concept of integrating the virtual and real realms. Firstly, we will delve into SDI, examining its distinct features, advantages, research status, and future developments in four pivotal aspects, following the trajectory of “real-to-virtual” integration. These aspects include:

• *Spatial Intelligent Sensing*, encompassing spatial sensing, spatio-temporal database management, and high-precision spatio-temporal mapping.
• *Data Transmission and Computing*, comprising 5G mobile communication, spatial high-performance computing, edge computing, and fog computing;
• *Intelligent Data Mining*, encompassing spatio-temporal rule mining, abnormality analysis, correlation analysis, prediction and decision-making;
• *Metaverse Space Construction*, involving metaverse model construction, multi-source model fusion, and metaverse model verification.

These technologies are systematically organized within the framework of “sensing-calculation-mining-construction”. Such a framework can be traced back to the early vision of smart cities [4], integrated sensors and electronics with databases, tracking, and decision-making algorithms. With respect to future trends and the current state [5], we further extend the four-layer framework by Tong et al. [6] which consists of Data collection, Data transmission, Data processing and Application, to cover more related technologies that help SDI harness data from real cities and effectively applies it within the context of the CM.

Simultaneously, the technologies encompassing the CM are systematically organized in a reverse “virtual-to-real” order, exploring the key components involved and their respective functionalities. The organization is as follows:

• *Smart City Simulation*, which encompasses spatio-temporal data visualization, spatio-temporal dynamic simulation, and digital twin city;
• *Human-computer Interaction* involves various modalities such as virtual reality, augmented reality, mixed reality, and brain-computer interfaces;
• *Distributed Technology* includes blockchain, Internet of Things (IoT), and Non-fungible tokens (NFTs);
• *Smart City Decision Support* incorporates concepts such as City Information Modeling (CIM), Virtual Geographical Environment (VGE), and urban middle platforms.

All these technologies are arranged in the order of “simulation-interaction-control-application,” emphasizing the progressive process of influencing the real world. As a newly developed concept, there lacks a widely accepted framework of the city metaverse. Therefore, we drew extensively on recent reviews. Among them, the inspiration to integrate digital twins with advanced technologies like IoT and blockchain serves as the backbone [7]. Valuable surveys in related fields like HumanComputer Interaction (HCI) [8] and VGE [9], [10] are used to fulfill the framework, and the very recent survey on the broad idea of metaverse [11] also inspired a lot. Ultimately, we believe it is appropriate to organize these related technologies in an order of increasing interaction degrees with the real world. By presenting the related technologies under this framework, we highlight capabilities in manipulating urban environments within the CM to ultimately impact real-world cities.

### 2.2 Relationship between spatial data intelligence and city metaverse

The proposed framework not only establishes a systematic order (ǣreal-to-virtualǥ and ǣvirtual-to-realǥ) for organizing the technologies, but also introduces a novel perspective for cross-domain comparison. For example, the 5G communication technology that facilitates high-speed data transmission in “Data Transmission and Computing”, also serves as the technology infrastructure for “Distributed Technology” including IoT [12] and blockchains [13]. Here, we adopt the context of “virtual-real integration” as the overarching framework, delving into the profound relationship between SDI and CM.

• *Smart City Decision Support & Spatial Intelligent Sensing*: Both areas are intricately linked to the physical urban space, representing the most tangible and “real” applications. Smart city decision support systems like urban middle platforms directly build upon the data collected by spatial sensing technologies [14], reflecting the direct collaboration between SDI and CM.
• *Distributed Technology & Data Transmission and Computing*: Both areas focus on the digital representations of physical space but with different emphases. “Data Transmission and Computing” cares about how data acquired in the real world can flow to the virtual realm, while “Distributed Technology” cares about how virtual information and instructions flow between physical devices [15]. Notably, the technologies involved in “Data Transmission and Computing”, such as 5G and cloud computing, provide the foundation of distributed processing technologies like IoT [12].
• *Intelligent Data Mining & Human-Computer Interaction*: Both areas are intimately tied to human behaviors, respectively exhibited in physical and virtual spaces. Relying on informatization and virtualization, behavioral patterns observed in the virtual and real domains can mutually inspire and complement one another [16].
• *Metaverse Space Construction & Smart City Simulation*: Both areas are directly associated with virtual city representations, showcasing the most distinct “virtual” characteristics. The digital twin city constructed through SDI serves as the foundational platform for CM simulations [17], and the outcomes of simulations can be utilized to calibrate the digital twin model.

The idea of linking SDI and CM stems from their interdependence and mutual promotion. We gain deeper insights into the intricate relationship within the framework by dissecting and comparing these four focus areas. This analysis provides an orderly framework and offers a novel perspective for cross-domain comparisons. Through this lens, we can examine the symbiotic relationship between SDI and CM, fostering a deeper understanding of their interplay and potential synergies.

The extensive data derived from SDI proves invaluable to the CM. Firstly, SDI supplies static statistical and dynamic application data of urban life, including population, buildings, traffic, and the environment, providing a foundation for constructing CM. Secondly, SDI facilitates intelligent management within the CM through analysis and processing. Thirdly, SDI aids in building refined models of CM with precise spatial data from real-world structures. Finally, SDI enables intelligent interaction within the CM through spatio-temporal visualization on terminal devices. Studies have sought to utilize SDI as a catalyst for advancing CM construction [204].

Conversely, the CM represents the possibilities offered by the virtual realm. Virtual representation of real space demonstrates the “virtual-real integration” and provides a promising approach to timely adopting urban changes for inclusivity and sustainability. Moreover, the CM furnishes SDI with a new and in-depth data source through refined urban models and vast virtual data for human behavior, simulation or generation. Virtual data facilitates the discovery of spatial data rules and allows for intuitive simulation and decision support of diverse urban scenarios, whose outputs can serve as inputs for iteration and evaluation of urban planning, transportation planning, and architectural design [18]. Furthermore, transformative technologies like VR and AR revolutionize the interaction between individuals and the city. Urban residents can be immersed in virtual experiences, transcending spatial barriers and fostering inclusive urban living [19].

In conclusion, SDI and the CM complement and reinforce one another. The construction of CM relies on the data and technical support provided by SDI, while the advancement of SDI benefits from the demands generated by the CM. Through their coordinated development, we can truly achieve the digital transformation and intelligent evolution of cities, enriching people’s lives with greater convenience and aesthetic appeal.

### 2.3 Method and data source

Despite significant advancements in relevant technologies, there is a noticeable dearth of literature and comprehensive reviews linking SDI with CM. In the field of spatial data intelligence, there are a lot of related reviews concerning spatial data. But they are either too early to cover the recent development of AI, such as Kopersk et al.’s survey on spatial data mining in 1994 [20] and Wang’s in 2005 [21], or not comprehensive enough, such as Du et al.’s review that focused only on machine learning algorithms, or Zhou et al.’s review [22] that targeted on 3D spatial data. Alsaedi et al. [23] provided a comprehensive review of the fundamental components and characteristics of big spatial data, which inspired our framework but still failed to bridge the SDI with the emerging metaverse. On the other hand, the CM field has some important surveys on the whole picture of metaverse technologies like Ning et al. [11], but have just started to examine their impact to the city [24]. Kusuma and Supangkat [25] reviewed the information technology that CM can use but did not provide a clear classification framework. Yaqoob et al. [26] reviewed the benefits, technologies and future opportunities of metaverse in smart cities, but did not include the role of smart-city-related technologies in promoting CM. It is very noteworthy that SE Bibri and Z Allam et al. carried out a series of studies [2], [27], [28], [29] that view metaverse as a virtual form of smart cities and analyzed the possible impact on platformization, governance, ethics and sustainability of cities, which strongly support our idea to bridge SDI and CM together. However, their series of work paid more attention to the influence of the social level and did not introduce the technology deeply enough.

It can be seen that the existing literature surveys fail to fully reflect the synergy between the SDI and CM. To address this gap, this paper adopts a critical literature review approach to provide a comprehensive overview of the field and various technological aspects. The primary focus of this review is to present an encompassing and informative perspective on the overall landscape and key research directions. The data utilized for this review primarily consists of journal papers and conference papers about the key technologies discussed in Section 2.1. Given the strong connection to industrial applications, grey literature such as white papers, technical reports, and government documents are also included. Notably, considering the emphasis placed on smart city development as a national strategy in China and the significant progress made in this domain, the literature surveyed encompasses publications in both English and Chinese.

## 3 Spatial data intelligence boosts city metaverse

### 3.1 Spatial intelligent sensing: data sources for virtual spaces

**Fig. 2:**
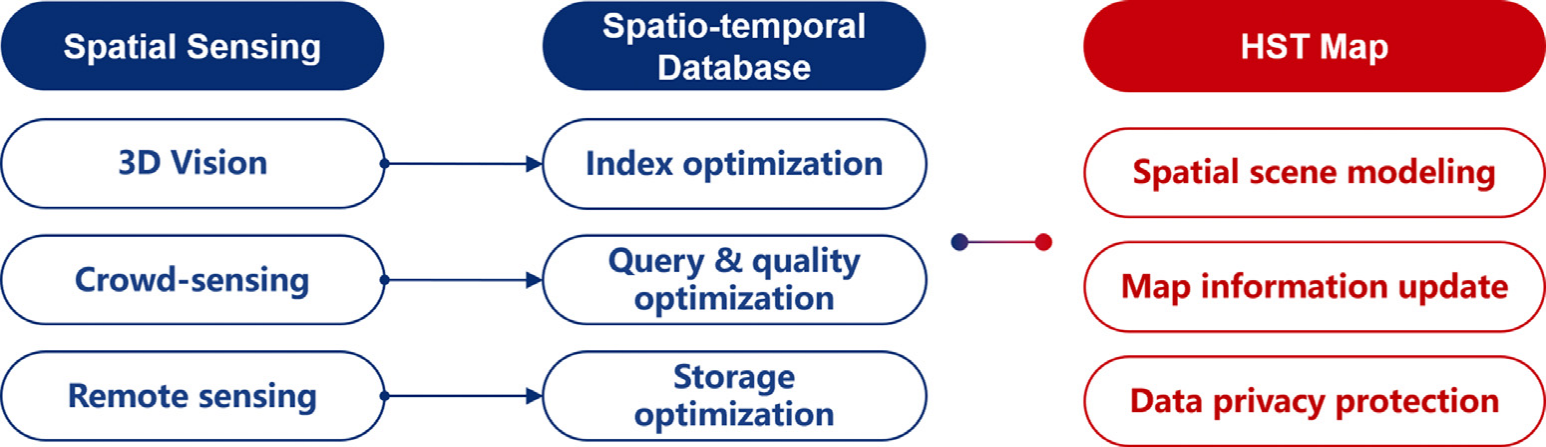
**Key technology involved in the review on Spatial intelligent sensing**.

Served as the initial step, spatial intelligent sensing encompasses the acquisition of spatial data using diverse technologies, subsequent data processing and storage for initial analysis, and the visualization of data on maps. Therefore, the subsequent sections will provide a detailed exploration of the three fundamental aspects: spatial sensing technology, spatio-temporal database technology, and high-precision spatio-temporal mapping (See Fig. 2). These technologies provide the basic data and storage management methods for subsequent SDI and CM work, emphasizing the extensive and efficient update of data sources. Their applications may be mentioned repeatedly in the other sections, especially in the space modeling of the metaverse, demonstrating complex synergies between different fields.

#### 3.1.1 Spatial sensing

The very first step of spatial intelligent sensing is to gather large-scale spatial data. Spatial sensing technology leverages a range of sensors, including aerospace satellites [30], aircraft and drones [31], smartphones and mobile terminals [32], smart wearable devices [33], industrial and household monitoring equipment [34], and wireless sensing devices [35]. Considering its application in urban scenarios, it is also known as “urban sensing”. As spatial sensing is a collection of broad technologies, we will mainly focus on several aspects that have made progress in recent years: 3D vision-based sensing, spatio-temporal data crowd-sensing, and remote sensing with intelligent interpretation.

• *3D Vision (3DV)* is a multidisciplinary field encompassing computer vision, computer graphics, and artificial intelligence. Its primary focus is utilizing vision sensors to efficiently capture and analyze three-dimensional information from the real world. In recent years, there have been significant advancements in 3D sensor technologies such as Lidar [36] and depth cameras [37] with the help of AI. Recent explorations in intelligent sensing systems [38], [39] have covered theoretical frameworks and important techniques, including 3D Simultaneous Localization and Mapping (SLAM), point cloud processing, 3D target detection and tracking, 3D scene reconstruction, and dynamic scene understanding.
• *Crowd-sensing of Spatio-temporal Data* refers to a technology that leverages distributed smart devices, such as smartphones, wearable devices, and IoT sensors, within a crowd to collect and share data about the urban environment and social phenomena. However, notable challenges exist from the distinctive characteristics of the data and sensing methods employed, including data quality, privacy protection, user incentive mechanisms, energy consumption, and data fusion paradigms [40]. As a result, current research endeavors in crowd-sensing are focused on the integration and analysis of the digital footprints left by large-scale crowds, to establish reliable and semantic-rich representations of group behavior across spatial domains [41]. The objective is to realize a mutually beneficial scenario for users, data providers, and application developers within the city metaverse [42], [43]. Addressing these challenges and advancing research in these areas will facilitate the full potential of crowd-sensing as a valuable source of spatio-temporal data in urban contexts.
• *Intelligent Interpretation of Remote Sensing* has gained significant attention due to the continuous advancements in higher temporal and spatial resolutions of captured imagery through satellites and drones. As a result, the demand for efficient processing and understanding of massive volumes of remote sensing data prompts extensive exploration of AI technologies [44]. Researchers have investigated into the mining of multi-source, multi-resolution, and multi-scale remote sensing data, trying to establish hierarchical scene analysis models [45] and interpretation techniques for ground and object information [46]. Besides, research on AI processing has been conducted in various fields including oceanography, land management, and transportation, to provide technical support for applications such as land and resources management, environmental protection, climate change analysis, and national security considerations [47]. These endeavors underscore the importance of intelligent interpretation methods and unlock their potential for diverse real-world applications.

#### 3.1.2 Spatio-temporal database

In the context of the three sensing methods discussed in Section 3.1.1, spatio-temporal databases are needed for storage, query, and optimization of collected data, which should effectively support the requirements of spatial and temporal data processing and analysis.

The spatio-temporal database is a technology designed to handle data with both spatial and temporal characteristics, including geographic information, meteorological data, traffic data, and more. In recent years, there has been significant progress in developing storage and query technologies [48], particularly in creating new spatio-temporal index structures that enable support for complex queries and fast retrieval [49]. Therefore, we will examine the advancement in index, query and storage technologies of a comprehensive survey.

• *Index optimization for 3D vision data*: Index optimization is crucial in achieving efficient data storage and querying due to the high spatial dimensions and complex geometric structures inherent in 3DV datasets. To address these challenges, spatio-temporal databases combine 3D index structures such as Octree, 4D R-tree, or KD tree with multi-scale representation and hierarchical storage techniques. These index structures consider the spatial and temporal attributes of 3D vision data, enabling the hierarchical description of spatial relationships [50], facilitating applications such as 3D point cloud data processing, spatial collision detection, moving object management, and trajectory data analysis. Furthermore, the spatio-temporal database incorporates specific topological data structures, such as the Half-Edge Data Structure, to support complex spatial queries related to the topological and spatial relations of 3D models [51]. Recent research has also focused on index optimization techniques based on deep learning methodologies [52]. By leveraging deep neural networks, high-level representations can be extracted, enabling the construction of more compact and efficient index structures.
• *Query optimization and quality management for crowd-sensing*: Optimization is critical for spatio-temporal databases. The dynamic nature of crowd-sensing data from many devices necessitates distributed storage and computing frameworks, such as Hadoop and Spark. To enhance query performance and reduce computational complexity, spatio-temporal databases employ techniques like grid indexing and trajectory clustering to reduce the query scope and streamline the computational workload. Given the potential for errors, noise, anomalies, redundancy, and invalid data in crowd-sensing datasets, data quality management is of paramount importance. Spatio-temporal databases implement quality inspection to identify potential issues [53], error correction to rectify inaccuracies [54], data reconstruction to enhance completeness and consistency [55], and quality evaluation to assess the overall reliability and usefulness [56].
• *Storage optimization for remote sensing images*: Storage optimization is another critical concern. Remote sensing images typically exhibit high resolution and contain abundant spectral information, necessitating specialized techniques for efficient storage and rapid access. Spatio-temporal databases employ block storage and pyramid structures to organize the image data into blocks and utilize hierarchical structures [57]. Similarly, compression and indexing technologies, such as band selection or PCA, are employed for spectral data. These techniques reduce storage space while improving query by selecting relevant spectral bands [58] or transforming the data into a more compact representation [59]. On this basis, performance evaluation tests encompass storage access, data division, index connection structures, and query algorithms to identify system bottlenecks and optimize efficiency. Recent advancements leverage machine learning to automate the optimization of system parameters [60]. By extracting data features such as frequency, basic unit count, and spatio-temporal distribution, a mapping matrix is established to represent the relationship between features and configuration parameters. This facilitates the creation of a trained system performance model for automatic optimization.

#### 3.1.3 High-precision spatio temporal map

Considering the particularity of spatio-temporal database, a very natural idea is to illustrate it on a precise map before further analysis, namely the High-precision Spatio Temporal map (HST map).

HST map refers to a digital map that provides an accurate representation of the earth’s surface environment and its temporal changes [61]. Distinguished from conventional maps, HST maps encompass information about the ground, underground, underwater, and air domains, enabling precise depiction of spatial alterations across different time intervals. Diverse sensor technologies including GPS, lidar, cameras, and inertial measurement units are used to capture relevant data, and advanced algorithms such as computer vision and machine learning are employed to process the collected information in time. The versatility of HST maps enables their utilization across various application scenarios like autonomous driving, traffic simulation and automatic parking.

While HST maps offer immense application potential, they also present significant challenges [62]. These challenges can be categorized into three main areas:

• *Spatial scene modeling*: The sheer volume of data makes efficient storage and retrieval challenging, and the processing complexity affects the performance of matching queries and semantic interpretation. Ensuring data quality and accuracy while managing the scale of the data remains a crucial challenge.
• *Map information update*: As the environment is constantly evolving, the ability to capture and reflect real-time changes in the map becomes crucial. The delay in updating map information can lead to discrepancies between the actual environment and the representation in the HST map. Ensuring timely updates to maintain the currency of the map information is an ongoing challenge.
• *Data privacy protection*: Protecting user privacy while maintaining data quality and timeliness is a complex task. Striking a balance between data privacy and the need for accurate and up-to-date maps requires robust privacy protection mechanisms.

Fortunately, technological advancements such as crowd-sensing, spatio-temporal data mining, and reinforcement learning have contributed to significant progress in addressing these challenges.

• *Spatial scene modeling*: The indoor-outdoor integrated scene modeling [63] focuses on the seamless integration of indoor and outdoor environments, involving techniques such as Multi-laser scanning simultaneous Localization and mapping (SLAM) techniques, extracting and matching the structures in both indoor and outdoor scenes [64].
• *Point cloud extraction*: Point cloud extraction aims to extract relevant information from the point cloud data. Efficient feature description algorithms can improve the effectiveness of feature extraction [65], while independent object extraction algorithms [66] and feature screening libraries automatically extract independent objects within the environment. Contextual feature extraction technology further enhances the accuracy and efficiency of point cloud processing [67].
• *Multi-platform large-scene fusion modeling*: This technology integrates data from multiple platforms to create a cohesive and detailed scene representation. Low-cost image sensors address blind spots and assist in scene reconstruction. Urban appearance modeling is then achieved through large-scale point cloud scene classification theory and the analysis of spatial topology relationships [68].
• *Map information update*: Begin with data collection using sensors, diverse local multi-agents data must be effectively fused to create a high-precision global map [69]. This requires establishing protocols and communication methods for data interaction between the data center and the agents [70]. Additionally, coordination and task assignment methods are essential to ensure efficient cooperation between agents and the timely completion of tasks [71].
• *Data privacy protection*: Data privacy protection involves several key technologies, namely differential privacy [72], encrypted computing [73], and anonymization processing [74]. Differential privacy safeguards individual privacy while preserving statistical characteristics by introducing noise during data release and queries. Encryption computing, including homogeneous encryption and secure multi-party computing, ensures secure data processing and transmission by performing computations on encrypted data. Anonymization processing protects privacy during data publishing and sharing by generalizing and suppressing sensitive information.

Despite the challenges faced by HST maps, ongoing advancements in technology are leading to the emergence of solutions and methods. These developments promise to enhance the quality, timeliness, and privacy of HST maps, thereby enabling the delivery of convenient, safe, and efficient services.

### 3.2 Data transmission and computing: operation guarantee of virtual spaces

Upon acquiring a large amount of urban data, the rapid transmission and computation of such data has arisen as a pivotal concern. Addressing data transmission encompasses not only the refinement of conventional communication technologies but also underscores the importance of 5G communication technology. On the other hand, the advances in data computation are characterized by two major efforts: the facilitation of high-performance computations for spatio-temporal data, and the architectural optimization through edge computing and fog computing paradigms. Henceforth, we take these three aspects as examples, considering their recent advancement and potential development in the future.

#### 3.2.1 5G mobile communication technology

Spatial sensing in Section 3.1 will continuously send back a huge amount of data from all city corners, which need fast, reliable and low-latency transmission. The emergence of fifth-generation mobile communication technology (5G) [75] has played a pivotal role in achieving it. The new architecture and technology of 5G offer higher transmission rates (with peak rates up to 20 Gbps), lower latency (in the millisecond range), improved reliability, increased network capacity, and broader coverage. The high speed and low latency of 5G provide support for data transmission, while the stability and reliability of the network ensure the seamless execution of data-related tasks.

• *Spatial Data Transmission*: Firstly, the high transmission rate of 5G enables rapid data transfer, facilitating real-time and high-precision applications within the city [76]. Secondly, the low latency of 5G is crucial for the timely collection, update, and analysis of real-time spatial data, particularly for applications like autonomous driving [77]. Thirdly, the enhanced network capacity of 5G supports a greater number of device connections, catering to large-scale CM systems that require continuous data collection from millions of vehicles and individuals in urban areas [78]. Fourthly, 5G incorporates robust security measures, including authentication, encryption, and secure communication protocols, ensuring enhanced data transmission security and privacy protection [79].
• *Spatial Data Computing*: Firstly, the substantial bandwidth offered by 5G technology facilitates the collaborative processing of extensive spatial data from multiple devices [77]. Secondly, the high computing performance and low latency of 5G enable real-time spatial data analysis, facilitating rapid processing within the CM environment. Thirdly, 5G supports cutting-edge technologies like edge computing and fog computing [80], [81], allowing spatial data to be processed and computed at the data source. This approach reduces data transmission costs and delays, alleviates the workload on centralized data centers, and enhances overall computing efficiency.

In conclusion, 5G technology significantly contributes to the transmission and computing of spatial data, offering essential capabilities for the operation of virtual space. Its characteristics provide ample assurance for the CM to deliver intelligent and efficient urban planning and management services.

#### 3.2.2 Spatial high-performance computing

**Fig. 3:**
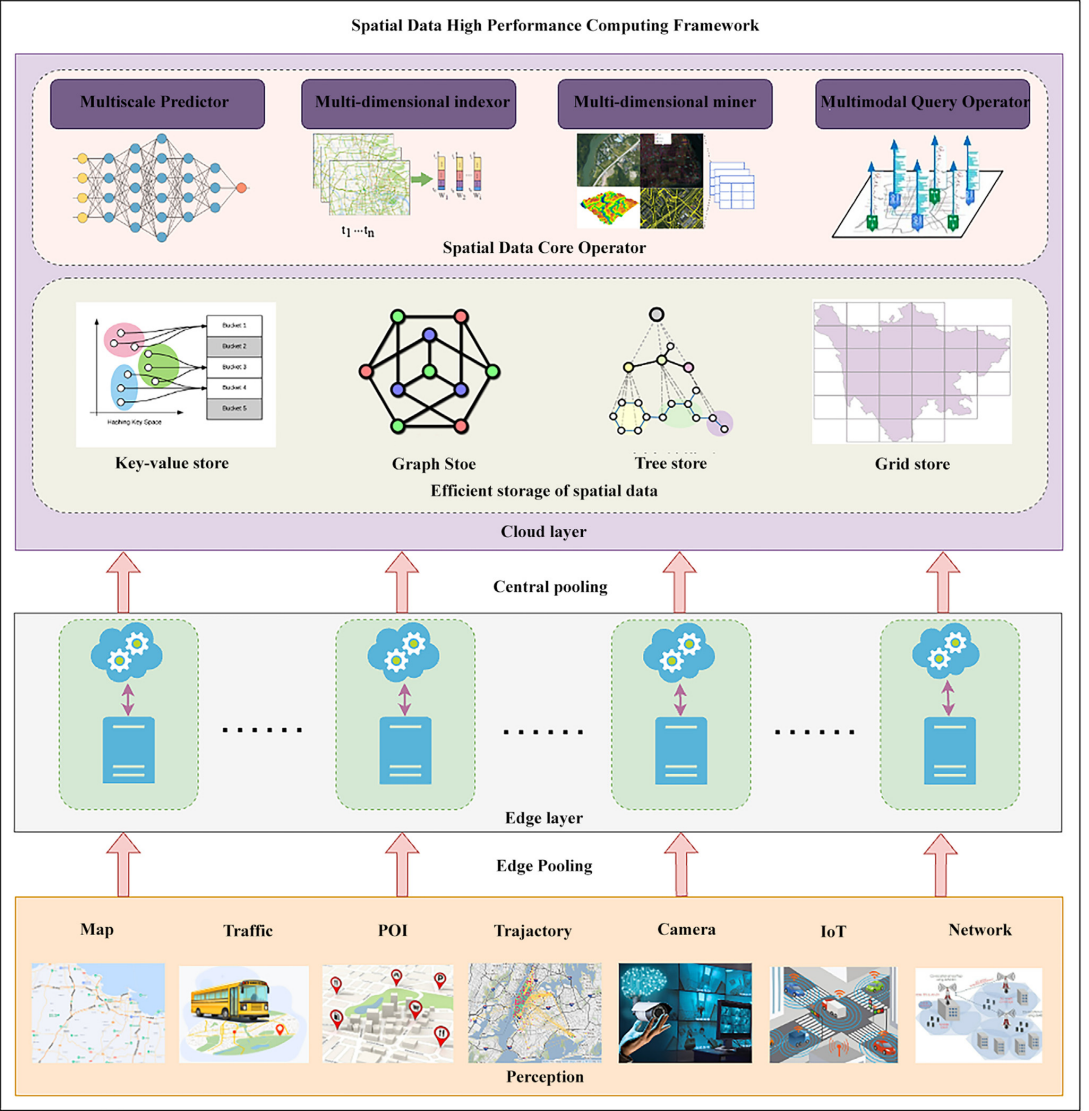
**Schematic diagram of high-performance computing framework for spatial data**.

The efficient computation of spatio-temporal representations is crucial for dealing with large-scale spatial data and its dynamic evolution over time. Through deducing the time-space state of people, objects, and things of the physical world in the large-scale cross-modal framework (see Fig. 3), spatial high-performance computing can accelerate many applications in the real world. Here we summarized the most common computational needs, including object search that searches nearest object in spatial databases [82], route query that searches shortest paths for navigation [83], and trajectory similarity that can be used in computational acceleration and clustering [84]. We selected these aspects because they are common but require computing resources and fast response, which motivate the algorithm to progress. There are also synergies between them since the index structure of objects can accelerate subsequent route queries, and the similarity calculation often relies on previous trajectory partition.

• *Moving Objects Search in Two-Dimensional Space.* How to realize searches for large-scale moving objects like cars and humans is an important topic of spatial high-performance computing. It can be roughly divided into moving object queries in Euclidean space and based on road networks. For moving object queries in Euclidean space, such as range query, k-nearest neighbor query, and reverse k-nearest neighbor query, the key issue is determining the search area containing the target moving object. Scholars have proposed various spatio-temporal index structures based on R-tree [85], Quard-tree [86], KD-tree [87], Voronoi, and Grid. On the other hand, the query of moving objects based on the road network is more complicated due to the need to calculate distance on the road network. In recent years, many works have studied the k-nearest neighbor query problem based on road networks, like SILC [88], ROAD [89], S-GRID [90], V-tree [91], G-tree [92], TOAIN [93], GLAD [94], TD-H2H [95], G*-tree [96], focusing on determining the target moving object under the premise of traversing as few road network vertices as possible. Besides, massive mobile objects and high concurrent queries challenge the storage and computing resources of a single computing node. Yu et al. [97] proposed a distributed index structure DSI and a distributed query algorithm DkNN, which can continuously split and merge according to changes in moving object density. MPR [98] proposed a concurrent execution mechanism for porting single-threaded query algorithms to multi-core servers. Furthermore, some work proposed GPU-based parallel query algorithms [99], which use GPU to construct the index and search the target area in parallel to generate a candidate result set.
• *Route Query in Road Network Space.* In real life, road network-oriented route query technology is an important demand of spatial data high-performance computing, which is essentially the shortest route query of the graph. Early typical work employed a heuristic incremental expansion like the Dijkstra algorithm [100] and A* [101], which use the greedy strategy to expand and visit the vertex. To further improve the efficiency, some work calculates and indexes the shortest distance between some vertices in advance like Contraction Hierarchies (CH) [102] which calculates the shortest distance bottom-up or top-down to form a hierarchical index. Based on the CH algorithm, relevant scholars have proposed the shortest route query algorithm based on the hub point label, such as HL (Hub Labeling) [103], DHP [104], PHL(Pruned Highway Labeling) [105], BHP [106], and SHP (Significant path based Hub Pushing) [107]. When facing huge graphs and high concurrent queries, it is more effective to use a parallel strategy. Pregel [108] is a distributed graph computing framework that supports graph parallel computing. Li et al. [109] proposed a distributed multi-modal route query algorithm on large transportation networks with three different categories and task instructions according to the query start point or end point. Aridhi et al. [110] used the MapReduce model to iterative divide the sub-graph for intermediate results. For the dynamic road network with ever-changing road transit time corresponding to edge weights, CANDS [111] created a distributed stream processing platform, adopted a divide-and-conquer strategy to divide the dynamic graph into disjoint sub-graphs, and proposed rules to minimize update operation caused by weight changes. Yu et al. [112] proposed a dynamic graph-oriented distributed top-k shortest route query algorithm, using the lower bound for the shortest distance between the sub-graph, and decomposing the original queries into local top-k query problems in multiple sub-graphs. Pedersen et al. [113] proposed a Time-dependent and Uncertain Contraction Hierarchies (TUCH) to support stochastic routing where the travel time of roads is not only time-varying but also uncertain.
• *Spatial Trajectory Similarity Calculation.* The trajectory data of people and vehicles is important for clustering the information from the physical world. For centralized calculation, directly computing on any two trajectories will incur a high computational cost. Therefore, pruning the search space by building an index structure is promising. Some work uses locally sensitive hashing technology to establish trajectory index and focus on the nearest k results [114], [115]. Grid index can also speed up the process through clustering grid cells for representative trajectories [116], or pruning in the time dimension with hierarchical grid index [117]. Besides, signature-based indexing like Strain-join [118] by Ta et al., dynamic space divisions like GeoSAX [119], global and local indexing scheme [117], [120] can also help to filter pairs of trajectories before distance computation, thereby reduce global transfer cost and local computation cost. To further improve the efficiency, distributed computing mode for algorithms is also important. The k-means algorithm can be optimized by Par3PKM [121] on the Hadoop platform, by Cui et al. [122] on MapReduce, and under the coarse-grained Dynamic Time Warping [123]. Similar to k-means, DPDBSCAN [124] proposed a distributed parallel clustering db-scan based on trajectory density partitioning. For similarity on the road network, DISON [120] was implemented on Spark with a two-level tree-structured global index, while Shang et al. [125] proposed a Spark-based system with a global index deployed on multiple computing nodes, which is similar to the work of Yuan and Li (Table 1).

**Table 1:** **Spatial high-performance computing algorithm**.

Algorithm	Task	Year	Key features
R-trees [85]	general index	1984	fast nearest neighbours queries
Quad-tress [86]	general index	1984	variable resolution
KD-tree [87]	general index	1975	fast queries and insert
SILC [88]	object query	2005	almost linear precomputing and storing
ROAD [89]	object query	2006	predetermined tree path to avoid costly network expansion
S-GRID [90]	object query	2007	pre-computed data independent of the data points
V-tree [91]	object query	2017	balanced search tree support dynamical update
G-tree [92]	object query	2015	assembly-based method for queries
TOAIN [93]	object query	2018	auto-tune shortcut-based index
GLAD [94]	object query	2019	scheduling algorithms to avoid conflicts and improve throughput
TD-H2H [95]	object query	2022	pre-computed weight functions
G*-Tree [96]	object query	2019	shortcuts between selected leaf nodes
DSI/DkNN [97]	object query	2014	distributed processing
MPR [98]	object query	2019	schedule query and update on the cores
G_grid [99]	object query	2018	GPU-accelerated with lazy update
Dijkstra [100]	route query	1956	breadth-first search for shortest path
A* [101]	route query	1968	heuristic of cost for shortest path
CH [102]	route query	2008	hierarchy node contraction and bidirectional search
HB [103]	route query	2002	distributed 2-hop covers of the shortest paths
DHP [104]	route query	2013	breadth-first search with pruning
PHL [105]	route query	2014	highway-based labeling with pruned
BHP [106]	route query	2014	ordering and compression of hub label
SHP [107]	route query	2017	heuristic path based ordering
PREGEL [108]	distributed route query	2010	graph parallel computing
CANDS [111]	distributed route query	2014	asynchronous answering and update
DTLP/KSP-DG [112]	distributed route query	2020	distributed for dynamic graph and insensitive virtual path
Li et al. [109]	distributed route query	2020	distributed on multimodal path
Aridhi et al. [110]	distributed route query	2015	parallel solve on subgraph
TUCH [113]	distributed route query	2020	time-varying, uncertain weight modeling and stochastic routing
LSH [114]	trajectory similarity	2004	locality-sensitive hashing
E2LSH [115]	trajectory similarity	2020	Geohash of domain POI and locality-sensitive hashing
SST [116]	trajectory similarity	2020	synchronously matching, grid indexing and query partitioning
Strain-Join [118]	trajectory similarity	2017	bi-directional mapping and signature-based similarity
MTSAX [119]	trajectory similarity	2018	GeoWard dynamic coding and trajectory partition
DISON [120]	trajectory similarity	2019	disjoint partitions by load balance and prune irrelevant
Tb-TS-Join [117]	trajectory similarity	2018	search space pruning and parallel processing
Par3PKM [121]	trajectory clustering	2015	MapReduce-based parallel three-phase k-means
Cui et al. [122]	trajectory clustering	2014	MapReduce-based k-means
Hu et al. [123]	trajectory clustering	2015	MapReduce-based coarse-grained Dynamic Time Warping
Wang et al. [124]	trajectory clustering	2017	distributed parallel clustering on trajectory density partition
DITA [125]	trajectory clustering	2018	global and local index with partition and cost-based balance

#### 3.2.3 Edge computing and fog computing

Besides the algorithms in Section 3.2.2 that accelerate computing, the optimization of computing frameworks, like edge computing and fog computing, is of special importance to issues such as high latency, low throughput due to uneven load distribution, and bandwidth limitations. As decentralized distributed computing technologies, edge computing integrates network, computing, storage, and application capabilities on a platform situated close to the data source, enabling the provision of services at the edge segment. On the other hand, fog computing is similar to edge computing but focuses on processing data in the fog computing layer closer to the edge devices. Currently, research in these areas is focused on the need for crowdsourcing and machine learning.

• *Spatio-temporal Crowdsourcing in Edge Computing.* With the rapid development of mobile Internet and the IoT, traditional crowdsourcing has developed into a new service called spatiotemporal crowdsourcing, also known as mobile crowdsourcing. Spatial crowdsourcing utilizes mobile Internet, online crowdsourcing platforms, and location services to connect crowdsourcing workers with temporal and spatial attributes in the real world, enabling crowdsourcing workers to actively or passively complete crowdsourcing tasks with spatio-temporal attributes, such as online taxi platform Didi and Uber. Using edge computing and fog computing technology to improve spatio-temporal crowdsourcing systems has become a research hotspot. Zhang et al. [126] proposed an edge computing-based Bidirectional K-Nearest Neighbor Crowdsourcing Allocation Protocol. Wu et al. [127] proposed a Weighted and Multi-Objective Particle Swarm Combination to optimize multi-objective task assignment. On this basis, Zhang et al. [128] proposed an Online Task Assignment across Regions based on Prediction algorithm with a two-stage graph-driven bilateral assignment strategy to solve the Cross-regional Online Task problem. Furthermore, edge cloud computing [129] unified with cloud computing in architecture and interface capabilities forms a complementary relationship and shows good performance.
• *Distributed Spatio-temporal Machine Learning.* Spatio-temporal data are usually provided by multiple service providers, which do not allow raw data sharing between providers. Therefore, data sharing and collaborative computing on the premise that data does not leave the local area inspire spatio-temporal federated learning with the concept of “computing moves rather than data move”. Spatio-temporal federated learning conducts model training on multiple decentralized edge devices and builds a general machine-learning model without sharing data. Space-time federated learning can be divided cross-device and cross-silo, with participants being edge devices (*e.g.* vehicle-mounted IoT devices and traffic flow monitoring sensors) and enterprises(*e.g.* service providers of shared bicycles and online car-hailing) respectively. Ye et al. [130] proposed a selective model aggregation method that individually trains local deep neural networks using local data at the edge devices. Tong et al. [131] discussed the Federated Range Aggregation (FRA) problems and proposed an efficient range-aggregation approximation model. And Zhang et al. [132] proposed federated adversarial domain generalization (FedADG) to equip federated learning with domain generalization capability. Furthermore, Hu-Fu [133] is a spatio-temporal data query processing system based on spatio-temporal data federation, which decomposes the processing of spatio-temporal query into plain-text operation and security operation. For online car-hailing services, Tong et al. [134] designed a federal learning-to-dispatch (Fed-LTD) framework, which achieves effective cross-dispatch by sharing the scheduling model and decision-making.

### 3.3 Intelligent data mining: a toolbox for characterizing virtual space

**Fig. 4:**
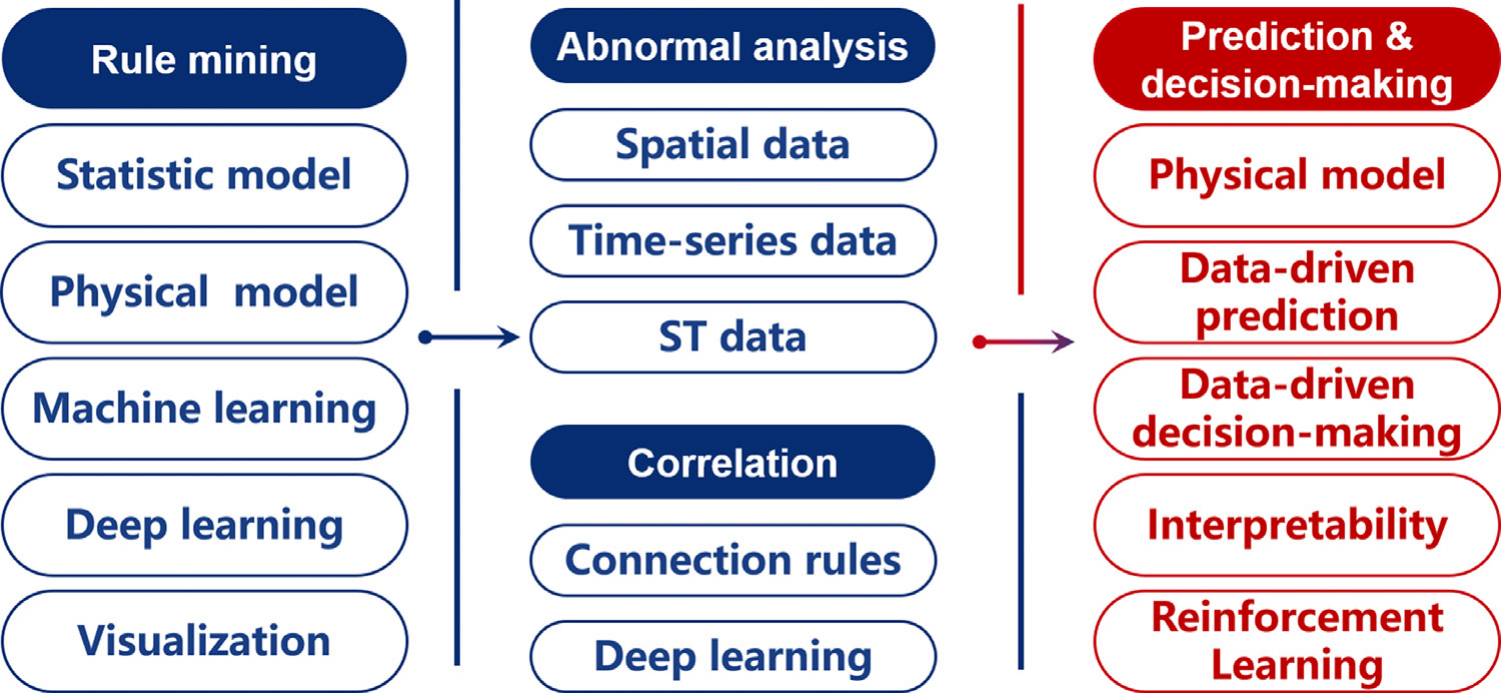
**Main research content of intelligent data mining**.

Based on the capability of spatial sensing, transmission, and computation, analyzing urban data to obtain valuable information and knowledge is possible. Spatio-temporal data can be classified into event data, trajectory data, reference point data, and raster data [135], each representing a kind of urban data with specific spatial and temporal characteristics. Considering such complexity among attribute features, spatio-temporal features, and correlation differences, leveraging the power of AI and machine learning, *i.e.* intelligent data mining, is of great importance. The core research areas of intelligent data mining, as illustrated in Fig. 4, involve the models and algorithms for rule mining, abnormal analysis, correlation analysis, and prediction and decision-making tasks.

#### 3.3.1 Spatio-temporal rule mining

Spatio-temporal data exhibits notable characteristics such as spatio-temporal correlation, multidimensionality, large volume, dynamics, and uncertainty. These properties pose challenges for data mining and call for utilizing AI and machine learning techniques to uncover patterns and extract valuable insights. The primary approaches for intelligent spatio-temporal rule mining include:

• *Traditional spatio-temporal statistical models*, such as the historical average and time series models [136] like moving average, ARMA, and ARIMA models, analyze and model temporal and spatial variables based on statistical principles and assumptions.
• *Domain understanding and physical modeling* involves physical process like levy flight [137], point process [138], or collective mobility model [139] in the modeling or clustering process [140]. By capturing the inherent causalities within the data, this approach enhances the interpretability of the derived insights [141] through methods including dynamic knowledge-based methods or knowledge graph [142].
• *Machine learning models* offer a range of techniques for various tasks in spatio-temporal data analysis. Classification methods, such as support vector machines and random forests [143], [144], are used for categorizing data into classes. Regression methods, including linear regression prediction and XGBoost [145], are employed for predicting numerical values. Clustering methods [146] encompass hierarchy-based, partition-based, and density-based approaches, identifying meaningful groups within the data.
• *Deep learning models*[147], particularly Convolutional Neural Networks (CNNs) and Recurrent Neural Networks (RNNs), offer advanced capabilities for spatio-temporal analysis. CNNs are well-suited for processing spatio-temporal data represented in image format, allowing for efficient feature extraction and pattern recognition. On the other hand, RNNs are adept at handling time series data, such as weather data, by capturing temporal dependencies and modeling sequential patterns. And the recent development of diffusion model have show great abilities in generate data that resembles the real world [148].
• *Data visualization*[149] is essential for effectively presenting spatio-temporal data in a visually intuitive manner, aiding in the understanding of their characteristics and dynamic trends. Heat maps are employed to depict the distribution of population density in cities, allowing for a quick assessment of population concentration. Contour maps, conversely, offer insights into terrain elevation and temperature variations, enabling the visualization of topographical features and thermal distributions.

#### 3.3.2 Spatio-temporal abnormal analysis

Unlike the mining task in Section 3.3.1 for the rule under normal circumstances, spatio-temporal data anomaly analysis aims to uncover the causes and assess the impact of anomalies, through identifying and analyzing entities that deviate from the expected distribution. Based on the combined space-time relationship, spatio-temporal data anomaly analysis can be categorized as follows:

• *Spatial data anomaly detection*: This method uncovers deviations from the universal spatial patterns in a small portion of the data, revealing the unique laws of geographic phenomena or processes. Representative techniques include the distance-based method [150], cluster-based method [151], density-based method [152], and graph-based method [152]. Spatial data anomaly detection aids in discovering abnormal points and patterns in spatial data during the construction of the CM.
• *Time series anomaly detection*: This approach identifies abnormal patterns in time series data by considering periodicity, trend, and randomness. Representative methods encompass statistical techniques (e.g., hypothesis testing, ARMA model, ARIMA model), similarity-based methods (e.g., KNN algorithm, LOF algorithm), and deep learning-based methods [153], [154].
• *Spatio-temporal data anomaly detection*: This method combines spatial anomaly detection with time series anomaly detection to identify spatio-temporal anomalies. Given the complex characteristics, diverse anomalies, and scarcity of samples, detecting anomalies in spatio-temporal data is challenging and heavily relies on machine learning and AI [155].

Anomaly detection techniques are valuable for identifying and understanding irregularities in spatio-temporal data, enabling proactive measures in the CM construction process. These methods enhance data quality, anomaly detection accuracy, and the overall reliability of the CM’s infrastructure and services.

#### 3.3.3 Spatio-temporal correlation analysis

In addition to addressing normal and abnormal circumstances, specific associations between entities, such as proximity or causation, are of great value. Spatio-temporal association analysis explores the dependencies and interactions among entities, delving deeper into the temporal changes and spatial interactions between objects, providing valuable insights for decision-making [156].

Traditional association analysis primarily relies on association rules, which first mine spatial association rules and then incorporate temporal association constraints, or vice versa [157]. However, these methods are conducted independently, neglecting the spatio-temporal coupling of data. Recently, the powerful feature learning capabilities of deep learning have been leveraged to automatically extract spatio-temporal correlation features from the data [147]. Consequently, recent studies have combined recurrent neural networks with convolutional neural networks [158] and graph neural networks [159], enabling simultaneous convolution operations on the spatio-temporal dimensions of the data.

By conducting spatio-temporal correlation analysis on real-world data, we can gain a better understanding of user behavior and activities in the city [160]. Incorporating spatio-temporal correlation analysis into the CM construction can create a more realistic, intelligent, and interactive virtual reality experience. This integration enhances the overall user experience and facilitates more applications.

#### 3.3.4 Spatio-temporal prediction and decision-making

Spatio-temporal prediction involves forecasting the future changes in geographical events or phenomena in both time and space. Spatio-temporal decision-making, on the other hand, entails selecting the optimal solution based on analysis results [161]. As the complexity of forecasting and decision-making continues to rise, traditional methods have struggled to meet the demands of decision-making processes. The advent of AI has significantly improved data processing efficiency, enhanced prediction accuracy, and enabled intelligent decision-making optimization [162]. Consequently, AI-based prediction and decision-making have become the prevailing approach [161], [163].

• *Domain understanding and physical modeling*: By amalgamating inherent domain insights and empirical laws, this approach fortifies the reliability and interpretability of predictions and decisions. By injecting the inherent comprehension of the domain’s intricacies into the transformative capabilities of AI, methods like differentiable decision trees [164] or knowledge graph [165] can yield more informed and contextually sound outcomes.
• *Data-driven spatio-temporal prediction*: Deep learning models like RNN and GNN possess powerful automatic feature learning capabilities in the spatio-temporal domain [147], which can be fine-grained to achieve accurately driving styles recognition [166]. They enable real-time dynamic analysis and prediction of massive heterogeneous spatio-temporal data, facilitating accurate forecasts of medium and long-term characteristics.
• *Data-driven spatio-temporal decision-making*: The visualization of spatio-temporal knowledge graphs is pivotal for decision-making capabilities [156], [167]. Using graph mining algorithms, valuable information is extracted and displayed through graph representation [168], such as the logistics decision optimization platform used by Cainiao [169].
• *Interpretable spatio-temporal prediction and decision-making*: Deep learning techniques alone lack interpretability. Spatio-temporal knowledge graphs integrate spatio-temporal data and knowledge to gain a better understanding of spatio-temporal rules [161], [165], [170]. Combining AI with knowledge graph technology greatly enhances interpretability and provides more insights, and Liu et al. [171] proposed a versatile UrbanKG for prediction and decision-making.
• *Deep reinforcement learning*: Deep reinforcement learning provides a path that has not been fully explored, that is, to directly model and predict spatio-temporal data through trial-and-error learning with agents. Using existing spatio-temporal data as expert knowledge, deep reinforcement learning can learn knowledge from data very effectively [172]. Other decision-making tasks includes traffic signal [173] or navigation [174].

### 3.4 Metaverse space construction: the entrance to the virtual space

**Fig. 5:**
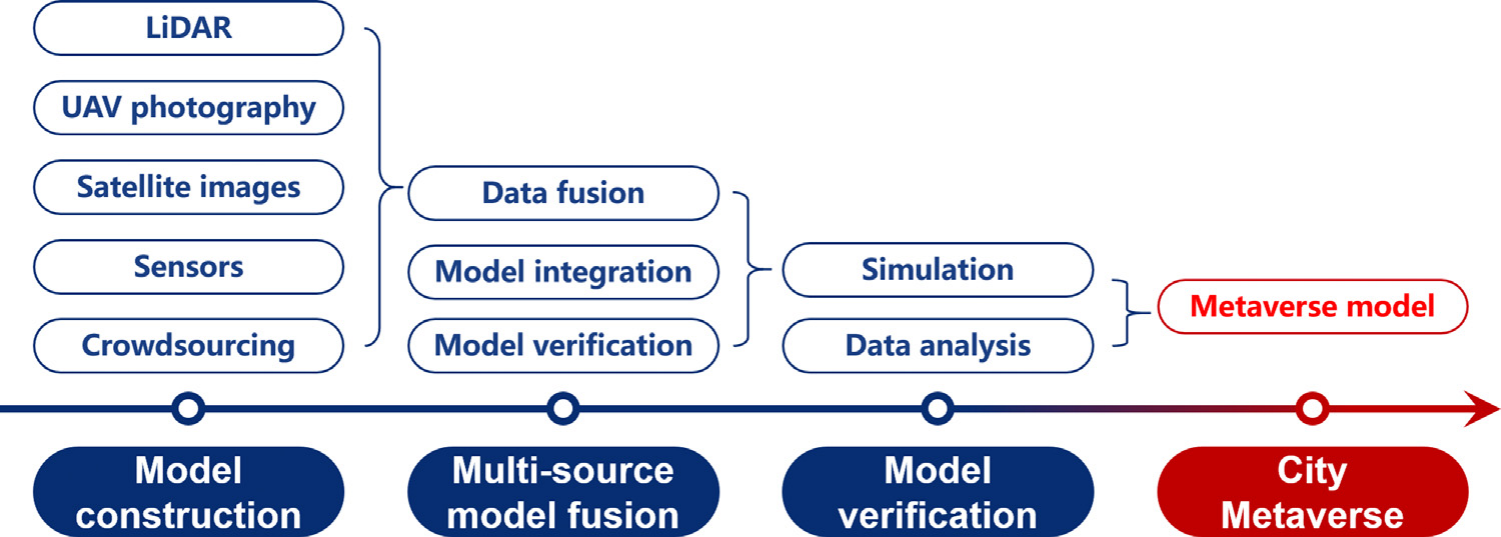
**Key technology involved in the review on Metaverse Space Construction**.

Completing the series of tasks to obtain information about the real city, one can finally construct a digital space in the virtual realm, which is nowadays called Metaverse. The construction of metaverse spaces serves as the gateway to the virtual realm, providing the foundation of immersive experiences, interactions, simulations, and augmented reality overlays. The metaverse space serves as the container of virtual content and the platform of virtual applications, which is expected to be close to real but easy to adjust. Here we discuss model construction, model fusion, and model verification technologies, following a sequential process to realize the CM model (See Fig. 5).

#### 3.4.1 Metaverse model construction

The first step of a CM is to establish a digital model, which finds applications in urban planning, simulation, and analysis. In recent years, the great abundance of data shifted research focus towards multi-source data. Souza L [175] provides a comprehensive overview of CM model construction technology, categorizing the current urban model construction approaches into the following categories according to their data source:

• *LiDAR model*: This approach involves acquiring 3D point cloud data of urban scenes using LiDAR technology. The data is then processed to extract features such as position, size, and direction, which are used to construct a city model [176].
• *UAV high-altitude aerial photography*: Utilizing UAVs equipped with visual recognition technology, high-altitude aerial photographs are taken to capture overlapping image areas. These images can be used for high-precision mapping and reconstruction [177]. For example, Google has employed this technology to build 3D city models in Google Maps.
• *Satellite remote sensing*: This approach involves capturing high-resolution remote sensing images through satellites. Advanced deep learning models [178] are now applied to extract ground object information, which is used to construct urban models.
• *Measuring instruments and sensors*: Various physical information about urban scenes is obtained using measuring instruments and sensors. For instance, the city government of Munich uses sensors to gather air quality data [179], which is utilized to simulate the city’s meteorological environment.
• *Social media and crowdsourcing*: This approach involves collecting urban scene information from the public through social media, mobile Internet, and crowdsourcing platforms.. As an example, researchers from MIT have developed Treepedia [180], which constructs models of green coverage in major cities worldwide using photos and location information uploaded by the public.

In summary, the selection of city model construction technology in the CM should be based on specific application scenarios and data sources. Each technology has its strengths and suitability for different purposes. Choosing the appropriate technology for a given application scenario is crucial in constructing the CM.

#### 3.4.2 Multi-source model fusion

Various data sources in Section 3.4.1 can all provide models at different scales and resolutions, therefore how to integrate them should be carefully examined. Multi-source model fusion integrates models from diverse data sources to create a unified CM model, aiming to leverage the strengths of different data sources for comprehensiveness and accuracy. The major approach includes the integration at the data level, the model level, or afterward verification.

• *Fusion technique based on data*[181]: This approach utilizes data fusion methods that consider factors such as weight, quality, or trust to integrate data from different sources. It aims to ensure the integrity and consistency of urban data by forming multi-level and multi-angle representations. For example, Jia et al. [182] achieved precise urban area extraction and model establishment by fusing multi-source remote sensing image data.
• *Fusion technique based on model integration*: This technique combines models from different data sources using similarity, association, and fusion rules. By leveraging these integration methods, the strengths of individual models can be combined to enhance the overall model accuracy. For instance, [183] employed a random forest-based model integration algorithm to accurately monitor changes in urban objects.
• *Fusion technique based on model verification*[184]: This approach involves using different verification criteria, such as accuracy or robustness, to evaluate the urban model. Various verification algorithms, including trust fusion, hierarchical fusion, and Bayesian network, can be utilized to assign weights and optimize multiple objectives.

#### 3.4.3 Metaverse model verification

Model verification technology plays a crucial role in assessing the accuracy and reliability of the established model from Sections 3.4.1 and 3.4.2.

One commonly employed method is simulation-based verification. By constructing a virtual urban environment and simulating its development process under different scenarios, the accuracy and reliability of the model can be assessed [49]. Furthermore, researchers can utilize Virtual Reality (VR) technology to present the virtual city to users, allowing them to experience and evaluate its practicality and feasibility. This approach necessitates a comprehensive simulation platform, along with various parameters and rules, serving as fundamental components for establishing and optimizing the CM.

During the simulation and verification process, various elements within the city can be simulated to observe their movement, interaction, and impact. By comparing and validating these simulations with real-world observations, the quality and accuracy of the City Metaverse (CM) can be further optimized. Simulation-based model verification methods have gained popularity in recent years. For instance, a study simulating traffic congestion in New York demonstrated that the CM could accurately reflect the actual urban traffic situation [185]. Another study verified the feasibility of shared transportation in reducing environmental impact by simulating factors such as air quality and environmental noise within the city [186].

Additionally, there are other model validation methods based on data analysis [187]. These methods rely on historical data to evaluate the prediction accuracy and applicability of the model. Such approaches are particularly valuable in traffic or climate models, where historical data can be used to validate the model’s effectiveness.

However, when applying validation methods based on data analysis, certain considerations must be considered. Firstly, the data quality is crucial, emphasizing the need for data cleaning and preprocessing. Secondly, selecting appropriate evaluation metrics should reflect the accuracy and applicability of the model. Finally, the analysis and interpretation of the results are essential to identify the strengths and weaknesses of the model, providing valuable feedback for refining the CM.

## 4 City metaverse empowers spatial data intelligence

### 4.1 Smart city simulation: a city running in virtual space

With the technical support of Section 3, we can extract and construct a metaverse space from a real city, and apply the power of digital technology to benefit our real world. The first step is to provide a virtual representation of the city and simulate various components and behaviors through smart city simulation. Such virtual representation, often called a digital twin, allows urban planners, policymakers, and stakeholders to analyze and optimize the city in a simulated environment.

**Fig. 6:**
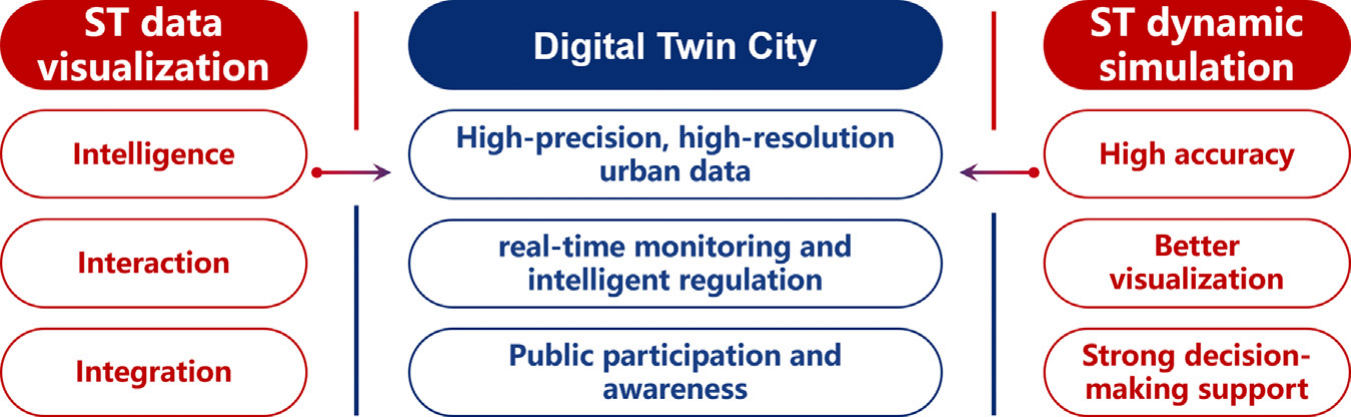
**Key characteristics of the advanced technologies involved in the review on Smart City Simulation**.

In the following sections, we will delve into the key aspects of smart city simulation, focusing on spatial-temporal data visualization, spatial-temporal dynamic simulation, and the digital twin city in order according to the depth of the simulation, which aims to create accurate and interactive virtual replicas of cities together (See Fig. 6).

#### 4.1.1 Spatio-temporal data visualization

Spatio-temporal data visualization, especially interactive exploration and dynamic demonstration, is the simplest form to present complex spatio-temporal data visually [188]. Due to its multi-source, multi-dimensional, interactive, and dynamic nature, it usually serves as the output of smart city simulation.

The main advantages of spatio-temporal data visualization technology include the following aspects:

• *Intuitiveness*: Spatio-temporal data visualization is designed to present abstract spatio-temporal data in front of users through intuitive visualization.
• *Interactivity*: Spatio-temporal data visualization supports independent selection of data dimensions, attributes, and granularity, enabling quick and accurate obtaining of the required information.
• *Real-time performance*: Spatio-temporal data visualization support displaying the spatio-temporal changes of data through dynamic demonstration to better meet the needs of real-time data analysis.
• *Globality*: Spatio-temporal data visualization can integrate spatio-temporal data from different sources to obtain more comprehensive and accurate data.

Currently, spatio-temporal data visualization finds applications in diverse fields, including map visualization [189], traffic visualization [190], and epidemiology visualization [191]. Advancements in sensor technology and wireless communication enable the acquisition and processing of large-scale, high-resolution spatial data. Looking ahead, spatio-temporal data visualization technology is expected to progress in the following directions:

• *Intelligence*: Emerging AI-based visualization technologies are expected to automatically process and analyze large-scale spatio-temporal data and be able to choose the best visualization form.
• *Interaction*: Advancement of HCI will enhance visualization interaction, and allow more free exploration through gesture recognition, speech recognition, AR/VR, etc.
• *Integration*: As the data from the city metaverse would be multi-source and multi-modal, effective and efficient data integration will become the key to spatio-temporal data visualization.

In conclusion, spurred by the demands of constructing and utilizing the city metaverse, spatio-temporal data visualization is set to undergo rapid development and innovation.

#### 4.1.2 Spatio-temporal dynamic simulation

Visualizing virtual cities alone does not provide enough insights that drive virtual cities. Spatio-temporal dynamic simulation [192] involves simulating various events in urban space, such as dynamic traffic, human mobility, urban disasters, and urban energy, which is the subsequent step for digital twins.

The key components of spatio-temporal dynamic simulation include establishing urban models, formulating simulation strategies, determining model parameters, conducting simulation experiments, and analyzing results. For detailed information on establishing urban models, please refer to Section 3.4. When formulating simulation strategies, consider factors such as the target of the simulation, spatio-temporal scope, and specific goals and requirements [193]. Determining model parameters is crucial and should be done based on experience or experimentation. Following simulation experiments, it is important to visualize, quantify, and analyze the results to draw meaningful conclusions and provide decision-making recommendations.

Thanks to the availability of vast amounts of data and advancements in AI, spatio-temporal dynamic simulation is experiencing remarkable improvements in accuracy and utility. Integrating AI algorithms with comprehensive datasets has unlocked new possibilities in simulating human mobilities within urban environments [194]. Deep learning models, in particular, have demonstrated exceptional capabilities in capturing and reproducing complex human behaviors [195]. These models leverage large-scale datasets to learn patterns and rules of mobility, enabling the simulation of realistic movements and interactions within a city [196], [197].

Moreover, the availability of high-resolution data has significantly contributed to our understanding of crowd behaviors in densely populated areas [198]. Analyzing this high-resolution data allows a comprehensive understanding of crowd dynamics [199]. Recent developments focused on capturing the daily trajectories and behaviors of individuals, which can uncover valuable insights into trajectories [172], [200], activity patterns [201], and the correlation between spatial and temporal attribute [148]. Integrating data-driven approaches, AI algorithms, and high-resolution datasets has revolutionized spatio-temporal dynamic simulation, enabling more accurate and detailed representations of urban dynamics. These advancements have far-reaching implications for urban planning, transportation management, and emergency response.

The advantages of spatio-temporal dynamic simulation lie in:

• *High simulation accuracy*: Based on actual data and physical laws, complex urban change processes like population migration, traffic congestion, and climate change can be simulated, which is of value to high-precision decision support for urban planning and management.
• *Better visualization*: The process of urban spatio-temporal dynamic change can be presented in simulation so that decision-makers and the public can have an intuitive understanding, which is of great significance in promoting urban democratization and transparency.
• *Strong decision-making support*: The urban change under different circumstances can be simulated to provide a comparison and selection of various schemes, thereby decision-makers can evaluate the pros and cons, and formulate more reasonable and effective strategies.

#### 4.1.3 Digital twin city

With the help of visualization and simulation, the digital twin city creates digital replicas of real cities for intelligent management, focusing on real-time synchronization with the actual urban conditions. It provides decision support for urban planning, design, operation, and management by updating, simulating, and analyzing real-time situations [202]. This technology is a significant trend in smart city research and finds applications in urban planning, traffic management [203], environmental protection [204], and disaster response.

The development of digital twin city technology has several advantages.

• Digital twin city can provide *high-precision, high-resolution urban data* that comes directly from the real-time collection during the operation, which are beneficial for decision-making management. And the simulation in a digital twin city can avoid potential risks such as unreasonable systems and models, thereby ensuring the authenticity of the simulation effect through interconnected urban systems.
• Digital twin city can realize *real-time monitoring and intelligent regulation*. With the help of AI models, real-time detection of road traffic flow can provide control of traffic signals. Similar methods can also be applied in environmental detection and crime prevention.
• Digital twin city can increase the *participation and awareness of urban residents*, which promotes urban democratization and community participancy. Digital twins in virtual spaces solve the problem of physical isolation of urban areas, enable a deeper understanding across different kinds of borders, and provide powerful tools for democratic urban governance.

It is important to acknowledge that the digital twin city technology still faces several challenges. Firstly, there is a need to enhance the quality and accuracy of urban data to improve credibility and realism. Secondly, the processing and computation of large-scale data pose challenges for real-time updates and evolution. Thirdly, ensuring data privacy and security, particularly with sensitive human data, must be emphasized before advancing the digital twin city.

### 4.2 Human-computer interaction: breaking the barriers between virtual and physical spaces

**Fig. 7:**
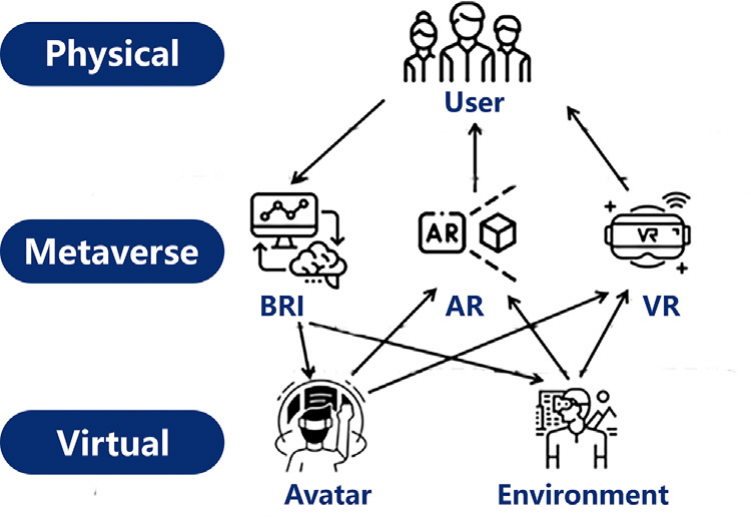
**Schematic diagram of Human-computer interaction for city metaverse**.

As a digital reality, CM can reconstruct and simulate the physical city. But without engagement with human interaction, the CM is neither self-completed nor adequate for practical use, highlighting the significance of HCI technology (Fig. 7). The HCI process comprises three main components: the real world, the virtual world, and the metaverse engine [205].

The real world component encompasses users who interact with the metaverse through devices, while the virtual world component consists of virtual characters and environments that respond to user input and exhibwit dynamic behavior. The metaverse engine facilitates interactions between the virtual and real worlds using technologies such as the brain-computer interface, augmented reality (AR), virtual reality (VR), and mixed reality (MR). In the following sections, we will delve into the concepts of VR, AR, MR, and the brain-computer interface.

#### 4.2.1 Virtual reality

Virtual reality (VR) is an immersive technology that combines computer graphics systems and interface devices to create interactive 3D environments. Using a VR device, the user’s visual range is expanded through a convex lens, while a gyroscope tracks the user’s head movements. The screen is continuously refreshed in real-time, allowing users to experience a 360-degree, three-dimensional space, resulting in a highly immersive visual environment [206].

Virtual reality is characterized by immersion, interaction, and imagination, achieved through its three main components: hardware, software, and content.

• *Hardware* encompasses input and output devices. Input devices can be hand-based or non-hand-based. Hand-based input includes VR handles, VR gloves, and gesture input devices, with handles being simple and easy to use, while gloves offer more advanced motion capture capabilities. Non-hand-based input includes eye tracking, motion tracking, and voice input. The primary output device is a head-mounted display that blocks the user’s sight to enhance the visual experience.
• *Software* creates virtual environments and objects to deliver an immersive experience. It can reflect the real world or create imaginary environments. Real-world reflection replicates existing physical environments, such as digital twins (see Section 4.1.3). Imaginary-environment creation involves painting, 3D modeling, or deep learning methods. Sound plays a vital role in setting the atmosphere and enhancing the sense of presence, complementing the visual scenes and objects.
• *Content* refers to the events, tasks, and experiences within the city metaverse, created by humans or machines. Deep learning systems have demonstrated their ability to represent and combine information hierarchically, which is crucial for generating content that closely resembles real-life experiences [207].

#### 4.2.2 Augmented reality

Unlike the full immersion of virtual environment in VR, Augmented Reality (AR) overlaps virtual objects onto real-world environments, including computer-generated images, sounds, 3D models, video, graphics, animation sequences, games, and GPS. AR supplements reality rather than providing an alternate reality, beginning with collecting real-world data through cameras and sensors. The user’s spatial position is continuously updated in real-time using cameras, gyroscopes, and other accessories. This information is used to calculate relative positions and fuse virtual content, resulting in a synthesized video presentation [208].

As a crucial technology in the city metaverse, AR blends digital visual effects with the real environment and is accessible through smartphones and other digital devices.

To support real-time AR operations, an efficient data transmission network like 5G (as discussed in Section 3.2.1) is essential. This network enables both precise and approximate AR services. Precise AR detects all potential objects for an immersive AR experience, while approximate AR focuses only on visually salient objects to reduce computation and communication overhead. Recent research proposes a self-adaptive AR services framework that adjusts to different network conditions and computing capabilities. The AR service provider allocates computing resources based on environmental information provided by AR users.

Object detection [205] is another important aspect of AR, as it requires accurate identification and localization of real-world objects for virtual object projection. Recent advancements in graph neural networks show promising potential in this area [209].

#### 4.2.3 Mixed reality

Combining the advantages of VR and AR, Mixed Reality (MR) merges real and virtual, aiming to create a unified space where real and virtual objects coexist and interact in real-time. The goal is to seamlessly blend the physical and digital worlds, allowing users to perceive and interact with both simultaneously.

**Fig. 8:**
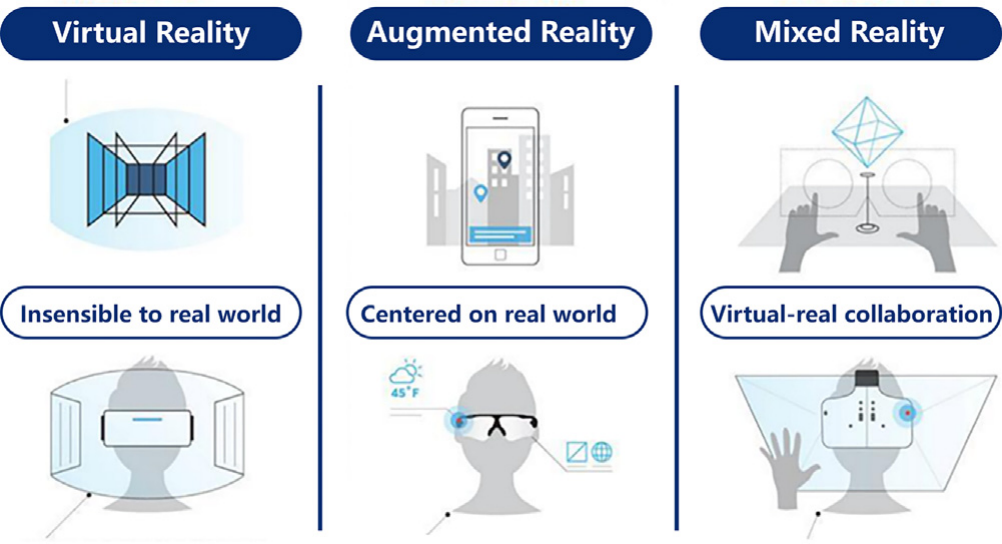
**Differences of VR, AR and MR**.

Virtual Reality, Augmented Reality, and Mixed Reality are related concepts with different display approaches (see Fig. 8). VR immerses users in a fully computer-generated environment, disconnecting them from the real world. AR overlays virtual content onto the real world, enhancing the user’s perception of reality. MR combines elements of both VR and AR, merging the real and virtual worlds and allowing for seamless interaction between them [210].

MR poses greater demands on perception and display compared to VR and AR, resulting in additional challenges such as model calibration [211]. Achieving precise calibration is crucial for MR technology, including accurate initial calibration of hand-eye coordination and effective real-time calibration to ensure long-term accuracy. Recent research [212] has introduced a real-time latent active correction algorithm for optical perspective and hand-eye coordination, which helps mitigate error accumulation in hand-eye calibration.

#### 4.2.4 Brain-computer interface

In addition to transmitting information through visual means like AR or VR, the Brain-Computer Interface (BCI) enables direct communication and information exchange between the brain and external devices. The process can be divided into three steps: detection, analysis, and control. Initially, specialized devices detect human brain activity, such as brain waves or magnetic fields. Subsequently, these signals are analyzed and processed to identify instructions from the brain. Finally, the extracted instructions serve as input for controlling devices, facilitating interaction between humans and computers.

BCI can be categorized into invasive, semi-invasive, and non-invasive methods [213]. Invasive BCIs involve implanting electrodes into the cerebral cortex, while semi-invasive BCIs place electrodes in the cranial cavity but outside the cerebral cortex. Non-invasive BCIs collect EEG signals using wearable devices attached to the scalp. Invasive BCIs provide accurate data but pose surgical and tissue rejection risks, whereas non-invasive BCIs avoid safety risks but capture weaker signals.

BCI holds promise as an *output* technique in the city metaverse. While conventional devices like speakers, headphones, screens, and VR/AR devices provide limited immersive experiences for hearing, vision, and touch, BCI can directly transmit images and sounds to the brain, enhancing auditory and visual interactions in real-time. Although currently used primarily in the medical field, BCI has the potential to shine in the metaverse.

BCI also enhances *input* flexibility by enabling users to control external objects through encoding and decoding brain waves. In an ideal scenario, EEG-based BCI allows users to control virtual characters in the city metaverse through thoughts and imagination. Existing applications such as the P300 speller [214] enable text input using EEG, and further research can enhance accuracy and efficiency.

BCI research has made significant strides, but it still faces major challenges. The primary challenge lies in accurately deciphering human intentions from brain signals, which are often characterized by a low signal-to-noise ratio. Brain signals are easily influenced by biological and environmental factors, and their non-stationary nature makes information extraction difficult. Although various preprocessing and feature engineering techniques in the time and frequency domains have been developed, they tend to be time-consuming and prone to distortion. Moreover, feature engineering heavily relies on human expertise, limiting generalizability. Although AI-based approaches have shown some progress, the dynamic nature of human thinking poses difficulties in precise classification. The city metaverse encompasses diverse and complex scenarios compared to medical applications, necessitating the development of new AI methods specifically tailored for BCI applications [215].

### 4.3 Distributed technology: intermediary connection of physical spaces

After engaging human interaction in CM, the need for physical-virtual synchronization emerges, aiming to reflect human behavior in the virtual world to every urban corner. Integrated distributed technologies, such as IoT and blockchain, provide such possibilities for CM. IoT enables the distributed collection and reflection of real-world devices, while blockchain provides data storage and security. Together, they support the operation and control of the real city in the CM.

**Fig. 9:**
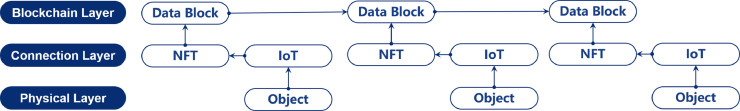
**Schematic diagram of IoT and Blockchain distributed technology**.

The conceptual diagram in Fig. 9 illustrates the integration of blockchain and IoT, consisting of the physical layer (IoT-enabled physical objects), the connection layer (digital representation generated through IoT and corresponding NFT), and the blockchain layer (storage of relevant information using blockchain technology).

#### 4.3.1 Internet of things

The Internet of Things (IoT) is a network that connects objects through information-sensing devices to enable their identification and management. In the context of the city metaverse, IoT technology plays a crucial role in collecting diverse data from urban sensors and delivering operation instructions to devices. Compared to 5G which is mainly a communication technology in Section 3.2.1), IoT focuses more on the heterogeneous, continuous, multi-dimensional, and multi-sourced connections between devices, which involves embedded devices, network architecture and application development. There is no doubt that 5G communication technology can greatly promote the progress of IoT, and such a close connection proves the synergy between SDI and CM.

The architecture of IoT can be divided into four main components:

• The perception layer, also known as the physical layer, includes sensors, actuators and other devices to collect various information from the surrounding environment. These devices then send the collected information to the network layer.
• The network layer, also known as the transport layer, forwards collected data from physical objects to information processing systems through wired or wireless means such as WiFi, Bluetooth, or infrared.
• The platform layer, is linked to a database and responsible for service management and data processing by technologies such as virtualization and cloud computing.
• The application layer, is the interface between the IoT system and users that provides intelligent and corresponding management for logistics, medical care, and urban construction.

IoT, as a distributed technology and a prerequisite for SDI, faces significant challenges in terms of security and privacy. At the perception layer, the transmission of multi-sourced information carries the risk of fake data and malicious tampering in public environments. To address these challenges, targeted algorithms and authentication mechanisms like Privacy-Preserving Data Publishing (PPDP) [216] are necessary. At the network layer, the efficient transmission speed exacerbates the problem of fake data, compatibility issues, and cluster security concerns due to the heterogeneity and complexity of the architecture. Secure routing protocols and data protection schemes are essential to mitigate these challenges and ensure the integrity and security of the IoT network.

Blockchain-based IoT provenance mechanisms provide a solution for ensuring data integrity and verifiability [217]. By recording all actions in the blockchain using a consensus-driven mechanism and digital signatures, transparency, immutability, and auditability are achieved. This technology has made a significant impact in industries such as supply chain management, where transparent and traceable records from production to sales are crucial.

#### 4.3.2 Blockchain

The concept of blockchain was introduced by Satoshi Nakamoto in 2008, through the article “Bitcoin: A Pemer-to-Peer Electronic Cash System” [218]. Blockchain is a distributed digital ledger that enables the recording and sharing of information within peer-to-peer networks, which provide authenticity and validity for IoT in Section 4.3.1 through a sequential chain structure and corresponding cryptographic algorithms. The features of blockchain have led to its widespread adoption in finance, information security, logistics, and manufacturing, which can be used as the extension of CM on real-world devices.

Blockchain technology encompasses different types of chains based on their degree of decentralization: public chain, private chain, and alliance chain. The public chain operates in a decentralized manner, where all network nodes participate and share information freely. However, it may have limitations in terms of scalability. On the other hand, the private chain is centralized, providing higher transaction efficiency and confidentiality. It operates under the control of a central entity. The alliance chain falls between the public and private chains, managed by multiple organizations working together.

In the context of Spatial Data Infrastructure (SDI), data plays a crucial role, and blockchain technology can contribute to its storage and security [1]. Spatial data often contains location information that may involve personal privacy or state secrets. By utilizing cryptography algorithms, the private chain or alliance chain can enhance information security and protect sensitive data. Furthermore, the blockchain’s ability to store time-series data facilitates auditing and retrospective analysis. It allows for the verification of results and decisions by tracing back through the recorded data, enabling the identification of issues in spatio-temporal data applications [219].

#### 4.3.3 Non-fungible token

**Fig. 10:**
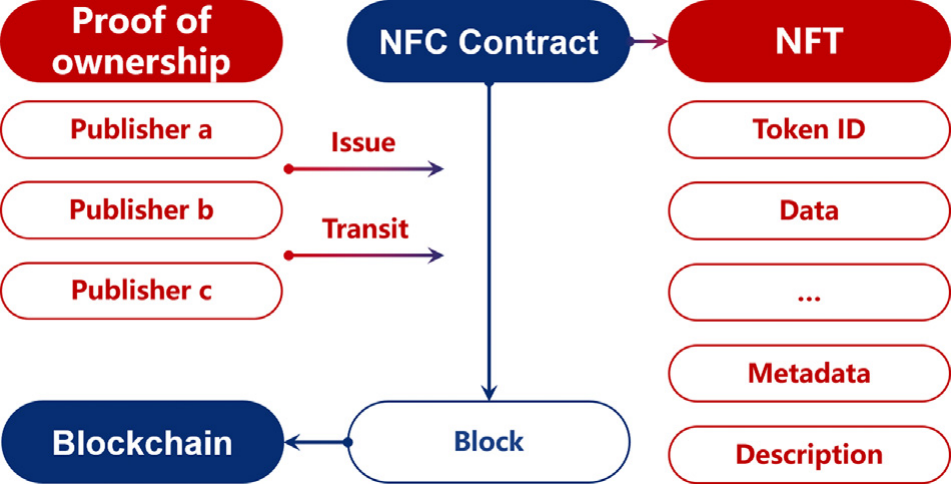
**Schematic diagram of the Non-Fungible Token**.

Non-fungible Tokens (NFTs) are unique tokens recorded on a blockchain ledger, representing certificates of distinct digital assets. In the CM, publishers convert digital items such as images, videos, and audio into NFTs, enabling users to freely trade these tokens through smart contracts on the blockchain (see Fig. 10). NFT provides advanced blockchain applications, goes beyond the original intention of information proof of device networks, and provides more possibilities for human behavior in CM.

As a new application of blockchain, Non-Fungible Tokens (NFTs) inherit and expand upon the characteristics of blockchain, including uniqueness, traceability, scarcity, and indivisibility. These features make NFTs suitable for use in the city metaverse, where they can serve as intermediaries for interaction and proof of ownership in the virtual world, as well as provide digital property protection. Given the diverse and heterogeneous nature of information in the city metaverse, NFTs can provide a credential basis to validate the contributions of owners.

However, despite the protection offered by blockchain, NFTs still face security and privacy challenges. Incidents such as those reported by SlowMist Hacked [220], which documented 56 security incidents in 2022 resulting in a loss of 65.43 million USD, highlight the importance of addressing these concerns. Ongoing research focuses on technologies like stealth addresses, zero-knowledge proof schemes, and reversible tokens such as ERC-721R [214], aiming to enhance the security and privacy of NFTs.

### 4.4 Smart city decision support: returning to the exit of physical space

After realizing smart city simulation, human-computer interaction and distributed control in CM, it will be a matter of course to integrate these technologies for decision-making support of smart cities [221], [222]. To achieve this goal, it is necessary to develop platforms or systems to carry the various modules from simulation, interaction and control. Here, we will mainly introduce the three kinds of platforms, namely the city information model, virtual geographic environment and urban middle platform. The three are closely related but conceptually distinctive, providing different emphases for different features and can be regarded as different solutions for CM.

#### 4.4.1 City information model

The City Information Model (CIM) is a digital representation used to describe and model urban spaces, infrastructure, environments, and social and economic systems [223]. It is derived from the Building Information Model (BIM), which focuses on digitizing the physical and functional characteristics of buildings [224]. The CIM, similar to BIM, integrates various data sources to provide comprehensive, accurate, and real-time information support for enhancing urban sustainability, livability, and competitiveness [225].

The CIM and the city metaverse are mutually beneficial as they share data, integrate technologies, and facilitate convergence. By leveraging the capabilities of the CIM, the city metaverse can use various models of prediction and simulation from digital twins to address digital inequality in planning and data fragmentation [226]. In return, the CM can enrich the CIM with immersive and interactive experiences, enabling better decision-making and urban management. The symbiotic relationship between the CIM and the city metaverse contributes to developing smart and connected cities.

• Data sharing: The CIM can provide abundant real-world data for the city metaverse, while behavior information generated in CM can also be fed back to CIM to provide new insights for urban management and planning [227].
• Technology complementarity: Both CIM and City Metaverse rely on advanced digital technologies, such as VR, AR and AI, which can achieve mutual support and collaborative innovation in different scenarios [228], [229].
• Application integration: CIM and City Metaverse can realize interaction and integration in urban planning, design, management and operation, like virtual urban planning experiments, public safety drills, emergency response training, etc.

On the other hand, the increasing evolution from digitization to intelligence gives rise to the concept of the city brain. The city brain is an AI-based application that leverages SDI and CM technologies to integrate diverse data sources for real-time monitoring, analysis, and decision support [230], [231], enabling intelligent, efficient, and sustainable urban development.

As the practice of large-scale artificial intelligence in the real world, the city brain can be viewed as an application of CM and CIM. On one hand, the city brain has five major application scenarios: urban traffic checkup, urban police monitoring, urban traffic micro-control, urban special vehicles, and urban strategic planning [232], all of which rely on advanced information and modeling technologies of CM. On the other hand, The visualization infrastructure of CIM [233], [234] provided a good platform for these applications’ usages of authorities and planners.

#### 4.4.2 Virtual geographical environment

Virtual Geographical Environment (VGE) is a new generation of geographic analysis tools for modern smart city systems [9]. It integrates several essential features, including geospatial analysis, geo-visualization, and geography-related planning and decision-making, as well as training, education, and entertainment. VGE reflects the early effort of CM in the field of GIS, focusing on opportunities for data support and functional expansion, and now benefiting from the technology advancement of CM [235]:

• Twin VGE: Twin VGE is developed based on the digital twin framework of metaverse, aiming to quantify the real-time and fidelity VGE with the constraint vectors and the attribute vector, enabling deep human interaction with the geographic environment [236].
• Geographical perspective: Traditionally, VGE provides three application levels, namely geo-object-based analysis, geo-process-based simulation, and multi-participant-based collaborative experiments. They can enrich the CM through knowledge collaboration, multi-person collaboration, multiple visualizations, spatiotemporal expression [237], which can provide a scientific basis for decision-making and management.
• More than one model: With the development of CM, it is now possible to catalog more than one model for any problem in VGE, with a human-centrally loop but focusing on different articulations of the applicability. The virtual infinity of the CM model defined new forms of VGE, which now forms the cutting edge of geospatial modeling and analysis [235].
• Immersive interaction: VGE can better represent our sense of place through the application of VR technologies [238], deepen our geo-experience from immersion to presence and further to embodiment [239]. Such immersive interaction can also benefit from recent machine learning methods like knowledge graph [240].

VGE has attracted researchers’ attention for more than two decades [241], firstly defined by Lin and Gong [242] as a sub-field of GIS and were designed for geographic understanding and problem-solving tasks by virtually augmenting users senses [9]. With the introduction of contemporary technology packages, including HCIs, distributed technologies, and simulations, VGE can now be seen as an application beyond CM [238]. With the continuous maturity of CM, VGE can enhance strategic visioning, pre-planning, public consultation, and traditional planning practices more [243], reflecting the vast possibilities in smart city decision support.

#### 4.4.3 Urban middle platform

The Urban Middle Platform (UMP) is an open infrastructure that aims to unify and coordinate urban management, services, and development. It provides efficient and convenient information services for the government, enterprises, and the public [244]. By establishing the UMP, data sharing, technology integration, service support, and intelligent decision-making can be achieved, promoting the digitalization, intelligence, and sustainable development of cities.

Compared to the city brain mentioned in Section 4.4.1, the UMP primarily focuses on data integration, technology integration, and an open platform, while the city brain emphasizes intelligent decision-making and automated management [230], [231], [232]. The UMP focuses more on the infrastructure level, providing unified data, technology, and service support across various urban fields, which can provide the city brain with necessary data and platforms when carrying out higher-level decision-making and applications [245].

The UMP and the CM can also be effectively combined. At the data level, the UMP collects and integrates various urban building data [246] to support the construction and operation of the CM, fostering innovation and collaboration. As a comprehensive platform, the UMP is open and scalable, enabling third-party developers and institutions to access it and provide rich applications and services for the CM [247]. The UMP can also facilitate the interconnection of the CM with other fields like education, supporting remote education and training in urban management, planning, and construction, and offering immersive learning experiences for students and professionals [248].

## 5 Application

SDI and CM can be extensively applied in various fields, and here we provide a concise overview of the eight most relevant application scenarios to highlight the current technological applications

### 5.1 National land survey and management

**Fig. 11:**
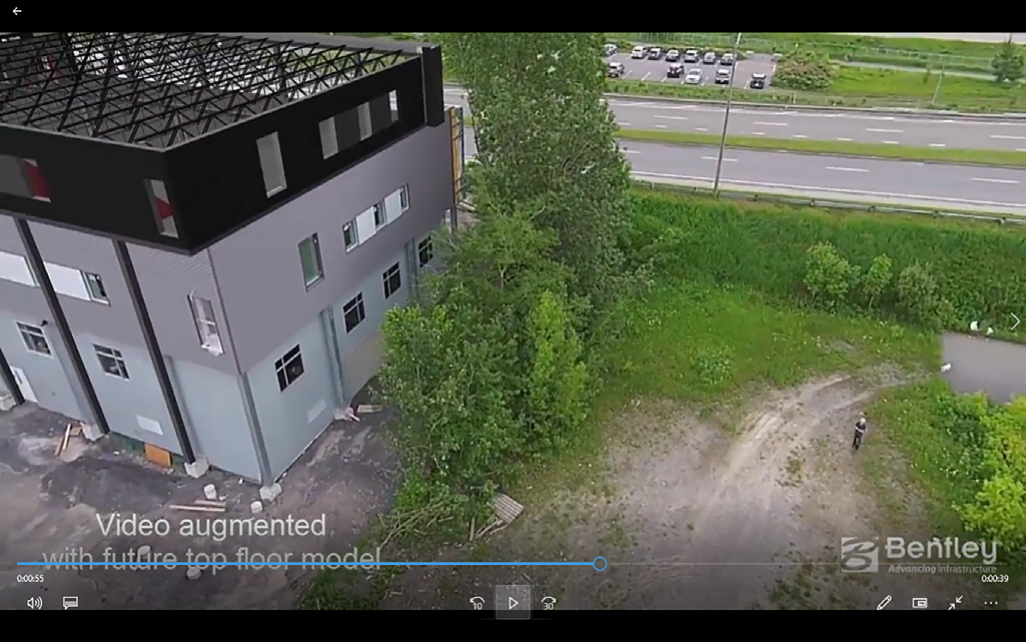
**The national land space planning scene supported by virtual simulation in city metaverse**.

Using 3D digitization and AI-driven knowledge bases in the CM, the potential impact of different planning schemes can be deduced to address various spatial land-use planning and management scenarios [249] (Fig. 11).

Establishing a real scene 3D surveying and mapping system serves as the foundational framework for Digital China and the development of the CM. In February 2022, the General Office of the Ministry of Natural Resources issued a notice titled “Notice on Comprehensively Promoting Real-Scene 3D China Construction,” which laid the policy groundwork for the application of the City Metaverse in national land management [250].

An exemplary application of the CM in national land space planning is demonstrated in the Xiong’an New Area. This region has pioneered an innovative approach known as the “integration of planning, construction, and management.” Leveraging technologies such as Building Information Modeling (BIM), Macro Geospatial Data (GSD), and the IoT, the Xiong’an New District’s CIM platform aggregates urban management-related data and adopts a micro-service architecture.

The platform encompasses the entire life cycle of a city, covering six stages: planning, construction, management, development, operation, and maintenance. It represents the first instance in China and the global context where digital city mapping and growth are seamlessly integrated. The implementation of this platform is expected to significantly enhance the development and refinement of Xiong’an City.

### 5.2 Low-carbon environmental protection

During the 14th Five-Year Plan, China aims to accelerate digital transformation to achieve “emission peak” and “carbon neutrality” goals, focusing on building a clean, low-carbon, safe, and efficient smart energy system. The combination of SDI and CM technology, facilitated by the digital twin platform, supports and drives the digital transformation of the energy sector [251].

Specifically, CM assists enterprises in low-carbon smart production in the following scenarios: First, SDI and CM enable efficient collection and analysis of production data and energy consumption data, facilitating timely adjustments for energy conservation and emission reduction [252]. Second, the digital twin simulation system allows for the rehearsal and assessment of different production tasks, leading to shorter decision-making cycles and cost reductions. Additionally, virtual production scenarios simulate processes and assess risks, aiding in crisis management and risk response training. Real-time data analysis using SDI provides early warning capabilities.

At a macro level, SDI and CM contribute to low-carbon smart city management. Utilizing multiple spatio-temporal data, refined energy demand forecasting optimizes energy demand and adjusts energy supply systems. By integrating supply and demand data from the energy industry with economic, social, environmental, and policy information, the virtual model in CM assists managers in optimizing and managing energy storage and transmission facilities for stable supply and efficient utilization. Virtual environmental simulation scenarios in CM enhance public understanding and experience of zero-carbon energy applications, promoting low-carbon lifestyles and reducing high-carbon emission activities like business meetings and travel.

### 5.3 Traffic planning

**Fig. 12:**
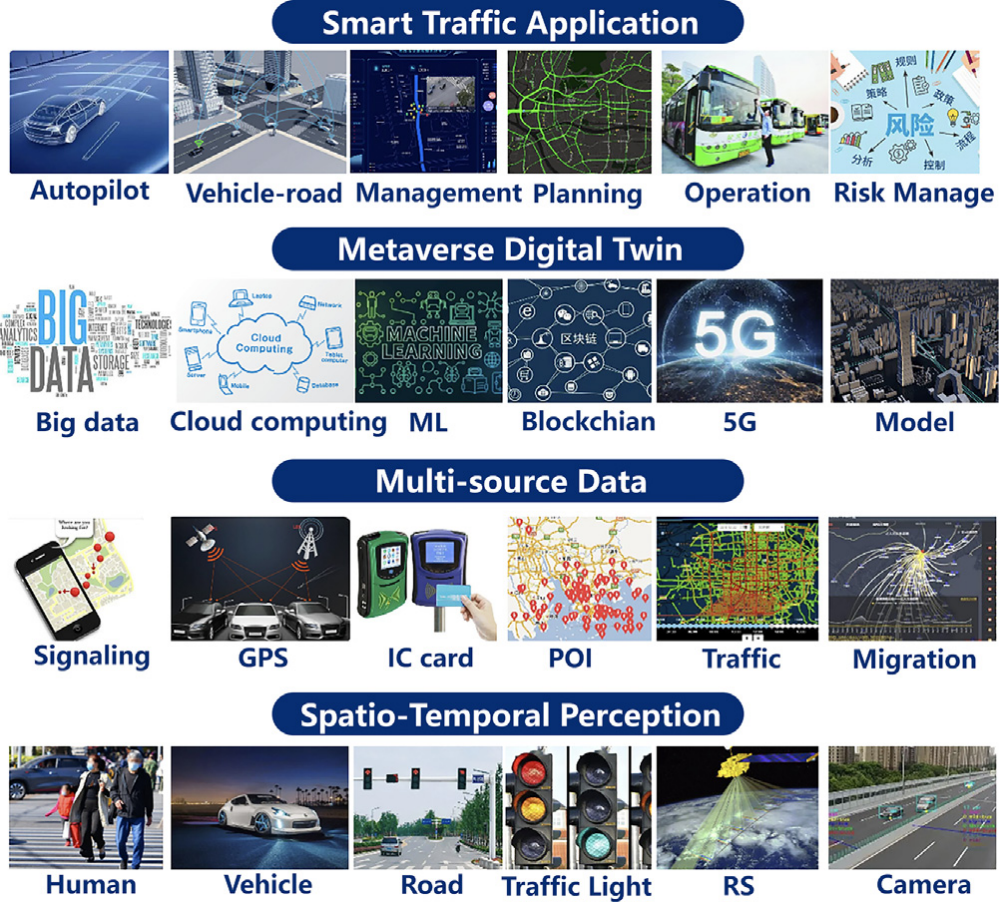
**Application of Spatial Data Intelligent Metaverse in Urban Transportation**.

The 14th Five-Year Plan prioritizes the acceleration of digital transformation in the transportation sector, advocating for the application of technologies such as big data, cloud computing, Internet of Things, and artificial intelligence in transportation. It aims to foster innovation and development of digital transportation [253]. The integration of SDI and CM creates a platform with diverse scenes, as depicted in Fig. 12.

• Real-time prediction and management: By collecting traffic spatio-temporal data such as urban road conditions [254], traffic flow, public transportation, and parking lots, a virtual model of urban traffic in CM can help to develop more effective traffic strategies. and provide an accurate and scientific basis for planning [255].
• Autonomous driving: Using SDI to obtain vehicles and environmental information in real-time can use to realize adaptive cruise control of vehicles, including adaptive acceleration and braking and steering.
• Vehicle-road collaboration: SDI can effectively perceive the information and status among urban vehicles, roads, people, weather, ground-air environment, traffic stations, traffic equipment and traffic events in real-time to realize the coordinated linkage of human-vehicle-road integration.
• Traffic planning and design: CM technology can simulate different traffic and planning schemes in the digital twin model to formulate more scientific traffic planning solutions [256], and ensure better solutions for sustainable traffic development [257].
• Public transportation operation management: SDI can use various sensors to collect and analyze real-time data of urban public transportation, and reflect these data in CM to provide intelligent decision support for the design and scheduling of urban public transportation, so as to improve efficiency and convenience of urban public transport.
• Risk management and resilience assessment: Through digital twin in CM, elements such as urban traffic network, roads, vehicles, passengers and traffic facilities can be accurately simulated for emergency response and traffic diversion strategies, providing a more comprehensive and accurate guarantee for the safe operation.

### 5.4 Cultural and tourism activities

The combination of SDI and CM enables the expansion of audience reach by offering virtual access to cultural facilities and tourism resources. By integrating spatial data, CM creates captivating and immersive experiences for tourists, thereby significantly enhancing cultural and tourism activities and driving the growth of the digital economy.

• Spatial data and CM technology enhance tourism by mapping attractions and creating virtual travel experiences, offering insights into tourist behavior for improved marketing and service strategies. In cultural activities, spatial data generates digital representations of heritage sites and landscapes, while CM enables participation in cultural events through VR and AR, making exhibitions more engaging and accessible.
• Urban spatial data includes information about the physical, environmental and cultural characteristics of the urban environment, which can be used to identify urban areas and map the boundaries of different urban functional areas, such as areas of natural or cultural significance. Detailed and accurate city maps can highlight the unique characteristics of each region thereby providing a decision-making basis for the tourism and cultural activities.
• SDI-based Point of Interest (POI) recommendations utilize user preferences, locations, and scenic spots to suggest personalized travel routes. Multi-source spatial data, including traffic patterns and user check-ins, predict visitor flows for effective merchant service recommendations. Real-time performance improvement while maintaining accuracy is a research focus.

### 5.5 Urban health

Rapid urbanization has led to health challenges like air pollution, unhealthy lifestyles, and an aging population. The “Healthy China 2030” Planning Outline and the New Urbanization Implementation Plan [258] emphasize the need for healthy, livable, and safe cities. The New Urbanization Implementation Plan of the 14th Five-Year Plan also specifically pointed out to promote the healthy, livable and safe development of cities.

Firstly, SDI aids in identifying urban health issues by analyzing spatial data. The urban built environment, including overcrowded housing, lack of green spaces, and tobacco and alcohol exposure, affects residents’ health [259], [260]. Intelligent algorithms combined with spatial data can calculate health indicators [261] and guide spatial improvements or suitable policies [262], [263].

Secondly, CM promotes medical equality between urban and rural areas. Through CM, medical knowledge and technology can be shared with remote regions, enabling online consultations and healthcare access for rural patients. And patients in rural areas can conduct consultations across spatial distances and receive online medical care.

Finally, metaverse technology facilitates health education and promotion in an accessible manner. It provides a virtual platform for public engagement, showcasing the benefits of healthy lifestyles, proper rest, nutritious diets, and clean environments, thereby fostering health awareness and encouraging real-life actions.

### 5.6 Resilient cities

**Fig. 13:**
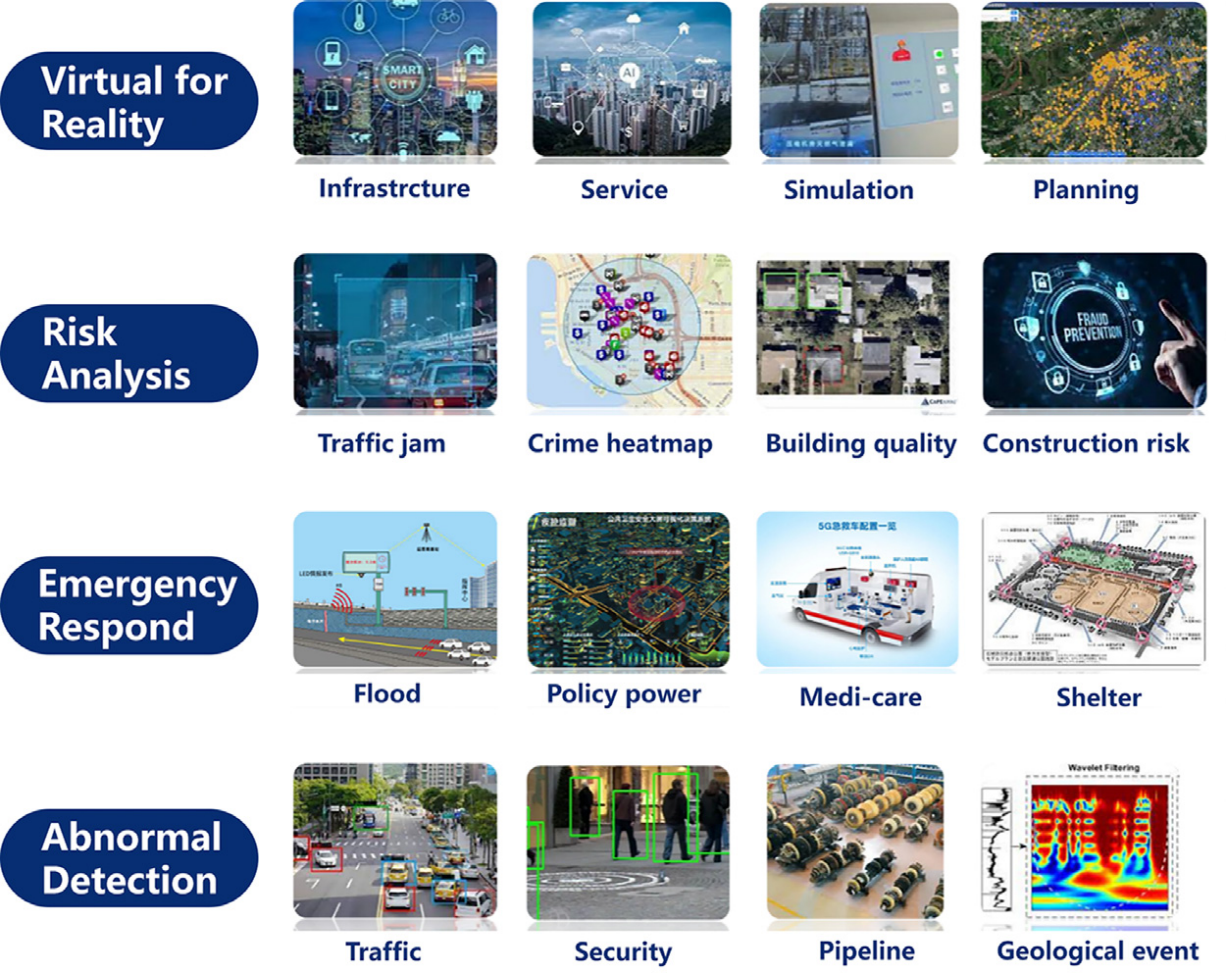
**Urban resilience improvement strategies based on city metaverse**.

Urban resilience encompasses a city’s capacity to withstand and recover from internal and external pressures by resisting, recovering, adapting, and transforming. SDI and CM have significant roles in enhancing urban resilience through the detection and assessment of abnormal events, optimizing resource allocation, and enabling intelligent emergency management (Fig. 13).

SDI plays a crucial role in detecting and evaluating urban abnormal events by monitoring and analyzing diverse data sources, enabling timely countermeasures and enhancing urban resilience. For instance, Lu Cong et al. achieved over 70% prediction accuracy in detecting urban traffic abnormal events using SDI [264]. Motta M et al. utilized SDI to monitor and predict urban floods, providing accurate flood warning information [265]. Wang Q et al. employed an advanced temporal graph convolutional neural network to accurately capture crime dynamics, optimizing police resource allocation [266]. Vancouver City utilized SDI and satellite images to monitor and analyze green spaces, identifying and utilizing underutilized resources to enhance the urban ecological environment [267]. Furthermore, the analysis of complex system empowered by deep learning can help to identified the dominate variables [268] and critical nodes [269] in urban systems.

CM enables the visualization, analysis, and optimization of potentially vulnerable areas and risks within a city. For example, Z Allam et al. developed a CM to dynamically simulate and predict urban transportation, energy, and the environment [2]. Y Han et al. proposed a hybrid evolutionary dynamics framework to provide consistent services in CM [270]. China Southern Power Grid utilized CM for multi-modal monitoring and early warning of the power grid, enhancing urban resilience through systematic transformation and dispatching schemes [271].

As urban resilience continues to develop, the scientific guidance of adaptation and transformation of various risks becomes increasingly important [29]. SDI and CM undoubtedly play pivotal roles in leading research, driving industrial development, and fostering practical innovation.

### 5.7 Urban supply chain

**Fig. 14:**
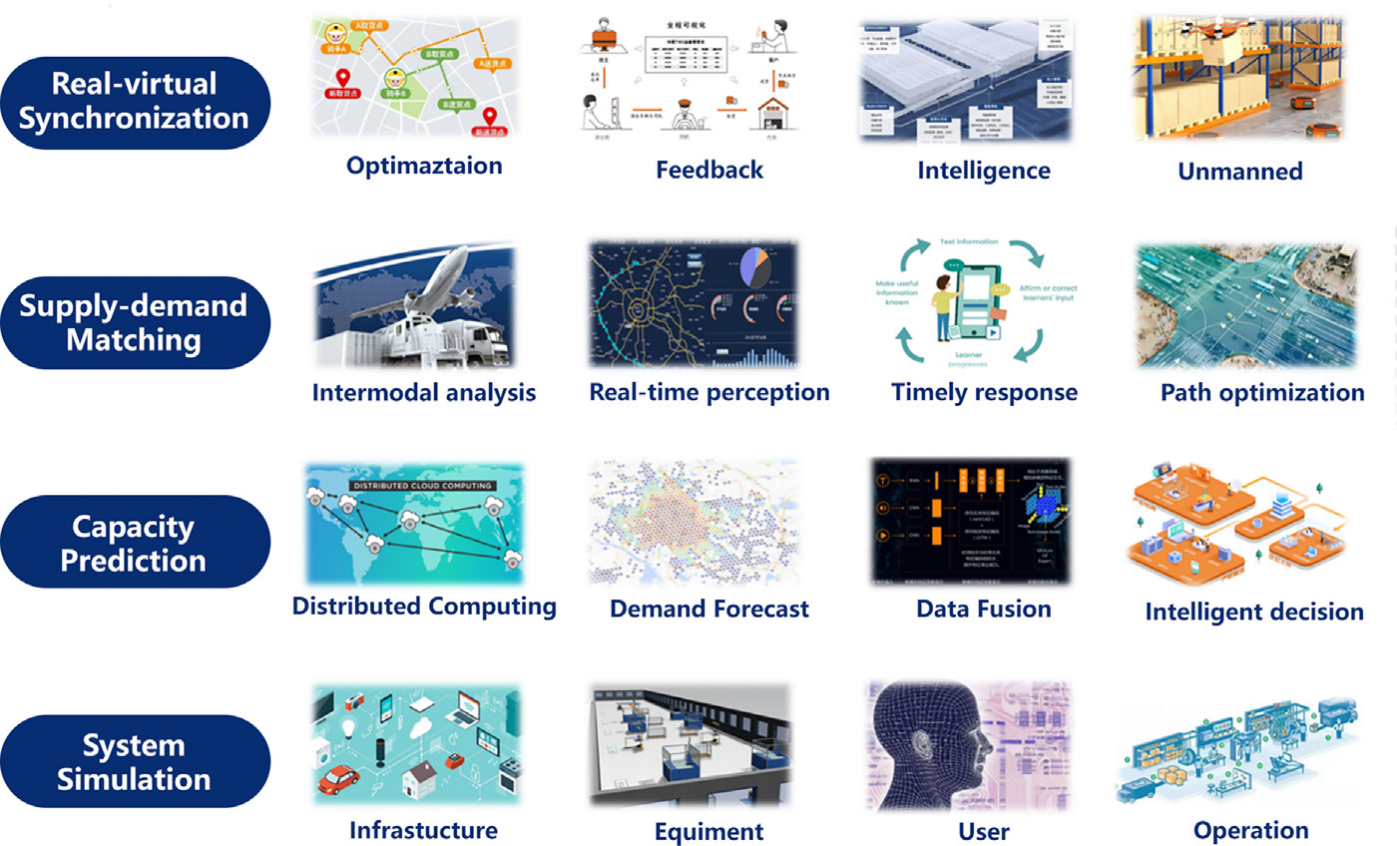
**The urban supply chain stack based on city metaverse**.

The urban supply chain, which encompasses the distribution of various materials and goods to urban areas, undergoes reorganization to accommodate mass customized consumption, digital retail, and instant response. To enhance user experience, the integration of the city metaverse offers visibility into the entire supply chain process and enables precise decision-making at a “second-level” (shown in Fig. 14). This promotes improved efficiency and adaptability in time allocation and spatial adjustments within the urban supply chain.

There are four major advantages involved:

• System simulation: By utilizing the digital twin model of infrastructure, equipment, users, and operations within the CM, it becomes possible to predict and intervene in events such as congestion, failures, and resource idleness in the supply chain. This enables proactive measures to address inaccurate planning, unreasonable plans, and uncontrollable processes, enhancing trust and effectiveness within the supply chain. CM applications like autonomous driving computing and intelligent warehouse systems have been successfully implemented in port logistics.
• Capacity prediction: Distributed computing can be employed to predict the total demand and transport capacity of urban logistics. Real-time capture of decentralized performance and order information allows for the integration of multi-modal data such as production planning, transportation monitoring, weather sensing, and IoT data. This significantly improves the prediction accuracy of the entire logistics link and enhances the timeliness of goods delivery.
• Matching of supply and demand: By simulating the behavior of the supply chain and considering the varying demand types and strengths of facilities in the city, it becomes possible to accelerate the adoption of multi-modal transport modes and establish suitable cargo loading and transport route selection. Real-time perception of external changes and immediate response ensure the provision of more matching facilities and resources, enabling intelligent optimization of logistics contract fulfillment paths and enhancing the responsiveness and flexibility of the transportation network.
• Synchronization of virtuality and reality: The CM’s perception, decision-making, and interaction capabilities are crucial for optimizing supply chain operations. The integration of distributed technology and spatial intelligence forms a real-time feedback system that helps improve efficiency, reduce operating costs, and continuously meet the demands of urban economic and social development within the urban supply chain.

## 6 Future direction

### 6.1 Cutting-edge technology

In the coming five to ten years, as related technical fields continue to advance and mature, the prospects for the technological development of SDI and CM will be extensive and boundless. In this discussion, we will explore the drivers of technological progress and prospects, focusing on the cutting-edge technologies that may play more important roles.

In terms of computational analysis methods, advanced deep learning will expedite the processing and analysis of spatial data, enhancing the accuracy and efficiency of intelligent management in CM [147]. We have witnessed the large generative model, such as GPT [272], LLaMA [273], Stable Diffusion [274], perform surprisingly well on tasks of text and images generation. These models leverage diverse urban data to generate images, voices, texts, or other outputs that resemble human performance, bridging the gap between the city metaverse and real-life scenarios. Recent work [148], [172], [275] has proved that generative models can be quickly migrated to spatio-temporal data for generating human trajectory, which is vital in SDI.

Meanwhile, the advancement in reinforcement learning which can learn from simulation environment [276] or expert knowledge [277] can accelerate the simulation in CM and delve into the simulation of human need [201]. Such a model can be utilized with large-scale decision-making models that effectively employ urban information in dynamic environments, supporting various real-life decision-making processes like traffic signal [278], power grid [276] or base station [252].

In terms of hardware architecture and technical support, high-performance computing technologies like edge computing and fog computing are progressively maturing. These technologies offer enhanced computational efficiency [279], greater storage capabilities [280], and improved support for the functioning of the city metaverse [281]. Those cutting-edge advance in computation offloading [282], energy consumption [283] or metaverse application [284] drive the progress of constructing a CM and promoting virtual-real interaction.

From the perspective of user access and experience improvement, virtual reality and augmented reality technologies will deliver a more immersive user experience in the city metaverse [285], [286], rendering urban planning and management more intuitive and interactive. With the recent development in hand tracking [287], eye tracking [288] and other intelligent technologies [7], commercial applications of VR/AR technology have already commenced addressing practical challenges such as user engagement, interface interaction, and experience enhancement.

In terms of security and privacy, blockchain technology will enhance data protection in the CM [289] and foster trust and reliability in digital cities [290]. As an accompanying security system for the city metaverse, blockchain advancement like decentralized mixing services [291], ring signature [292], non-interactive zero-knowledge proof [293], or homomorphic cryptosystem [294] stimulating new avenues for technological development and innovative applications.

Besides those technologies advances, soft power such as data sharing and open cooperation will serve as crucial driving forces for advancing the CM [295], [296]. Collaboration among government, enterprises, and academia in building and utilizing digital city infrastructure and resources will be pivotal, which should be carefully examined on the aspect of policy [297]. Technological progress in differential privacy is indispensable for promoting the openness [298], contributing to the future intelligence of spatial data and the overall growth of the CM.

### 6.2 Future of industrial chain

SDI and the associated industrial chain of the city metaverse act as catalysts for the coordinated development of various disciplines and industries across different sectors. The construction of SDI relies on substantial infrastructure support from upstream industries such as computers, network communications, sensors, the IoT, and cloud computing. Furthermore, it extends to the manufacturing and operation of space data acquisition equipment like drones and satellites, as well as SAAS providers offering data processing and analysis services. Similarly, the development of the CM involves upstream industries related to information technologies, including professional city modeling, virtual reality, and data visualization. Additionally, it extends to emerging sectors like artificial intelligence and autonomous driving. The downstream applications of SDI and the city metaverse are extensive and have been discussed in Section 5.

Moreover, the application of SDI and CM collaborative development among different industries and fields. For instance, employing SDI for logistics trajectory monitoring and optimization enhances the efficiency and service quality of urban logistics, thereby fostering the development of related industries such as e-commerce and manufacturing. By extending the industrial chain of urban informatization based on CM [299], the formation of industrial clusters interconnected becomes a significant transformation for future urban development.

Over the next five to ten years, the technological development of SDI will be closely intertwined with the construction of the CM. This synergy will facilitate the digital transformation of cities and the establishment of smart cities, encompassing infrastructure, software and hardware equipment, services, and other aspects of technological development. The ultimate goal is to create smarter, sustainable, and livable urban environments for the benefit of all.

### 6.3 Legal, regulatory and ethics

The technological progress and development of SDI and the CM hold immense potential, but they are also accompanied by legal and regulatory challenges. It is crucial for the government to enhance supervision over the city metaverse, establish clear legal frameworks, identify responsible entities, and define industry standards. This can be achieved through the implementation of laws, regulations, and guidelines. Furthermore, regulatory authorities should improve their enforcement capabilities and technical expertise to effectively oversee the city metaverse.

In addition to government supervision, CM service providers have a responsibility to self-regulate their operations. They should take proactive measures to ensure the safe and stable functioning of the city metaverse. This involves implementing robust security protocols, adhering to privacy regulations, and adopting best practices in data management. By prioritizing self-regulation, CM service providers contribute to the overall safety and reliability of the city metaverse ecosystem.

Addressing legal and regulatory challenges is vital to foster a trustworthy and responsible environment for the development and operation of SDI and the city metaverse. Collaboration between the government, regulatory authorities, and CM service providers is essential in establishing a framework that promotes innovation while safeguarding the interests of individuals and society as a whole.

• The legal status of the city metaverse remains ambiguous, posing challenges in terms of management and regulation. As the city metaverse encompasses various economic activities, there is a need to establish clear legal frameworks that address virtual property rights, privacy protection, and network security. It is essential to develop comprehensive laws that define the legal nature, transactions, and taxation of virtual property, while also addressing concerns such as money laundering within the virtual space.
• Effective management and security of data within the city metaverse present significant challenges. It is necessary to develop appropriate technical solutions and regulations to mitigate the risks of data breaches. Regulatory authorities should focus on ensuring that service providers inform users about the scope of data usage, establish robust consent mechanisms, and implement stringent self-regulatory procedures to safeguard data privacy.
• Governance of the city metaverse requires coordination among various sectors and international cooperation. The governance of the city metaverse mirrors that of a physical city, involving multiple departments and agencies. Lessons from governing real cities can inform the governance of the city metaverse, but adjustments tailored to its unique nature are necessary. Additionally, given the transnational nature of the virtual space, it is essential to foster cooperation and coordination among different countries and regions to address regulatory challenges that extend beyond national boundaries.

Besides, in the ideal future created by the symbiosis of the real world and the digital world, there are significant security risks and ethical issues that need to be addressed in the city metaverse:

• Technical reliability: While advancements in information technologies have improved security measures, the city metaverse is still susceptible to natural disasters, human errors, and cyber threats. Failures of smart devices, system vulnerabilities, and attacks on software systems can disrupt operations and compromise the integrity of the entire city metaverse.
• Data privacy: The use of spatial data intelligence technologies in the city metaverse can lead to privacy breaches. Existing privacy protection measures are limited in dealing with complex relationships in urban spatio-temporal data. More efficient and comprehensive approaches are needed to address data privacy concerns.
• Ethics and morals: The city metaverse reflects and extends the real world, introducing complex ethical and moral challenges. Virtual avatars and interactions in the metaverse can lead to behavior changes and raise questions about morality and legality. Ensuring ethical standards and preventing harmful behaviors within the metaverse is crucial.

To effectively address these security and ethical issues, collaboration among governments, enterprises, and the public is necessary. The government should provide oversight and regulation while remaining open and cautious. Enterprises should proactively assess and mitigate risks through scene examination, algorithm design, privacy protection mechanisms, and compliance reviews.

## 7 Conclusion

In summary, we have organized the research and technologies related to the CM into a coherent framework. Firstly, we explore SDI technologies that enable the collection of real-world information for constructing a virtual CM. Next, we discuss the integration of these technologies with SDI to enable interaction and management of real cities from the virtual perspective. We also highlight the practical applications and potential of these methods in real-world scenarios. Finally, we address the new challenges in technological progress, the industrial chain, legal and regulatory aspects, as well as ethics and morality.

The CM relies on comprehensive and accurate data provided by SDI to create urban models and support urban planning, construction, and management. Only with sufficient, diverse, and reliable data can the CM achieve a more realistic, refined, and immersive experience. Additionally, the development of the CM can drive advancements in SDI. Technologies such as virtual reality, augmented reality, and artificial intelligence that are utilized in the CM present new opportunities and application scenarios for SDI, enhancing our understanding of spatial data intelligence. For instance, AI applications within the CM can foster innovations in areas like smart transportation and environmental protection. SDI requires an open, inclusive, and rapidly evolving platform like the CM, which transforms our perception of urban spaces into actionable intelligence that impacts urban operations. We believe that the integration of SDI and the CM represents the main direction for future development in this field.

## Declaration of competing interest

The authors declare that they have no conflicts of interest in this work.
